# Progressive resistance training for children with cerebral palsy: A randomized controlled trial evaluating the effects on muscle strength and morphology

**DOI:** 10.3389/fphys.2022.911162

**Published:** 2022-10-04

**Authors:** Britta Hanssen, Nicky Peeters, Nathalie De Beukelaer, Astrid Vannerom, Leen Peeters, Guy Molenaers, Anja Van Campenhout, Ellen Deschepper, Christine Van den Broeck, Kaat Desloovere

**Affiliations:** ^1^ Department of Rehabilitation Sciences, KU Leuven, Leuven, Belgium; ^2^ Department of Rehabilitation Sciences, Ghent University, Ghent, Belgium; ^3^ Faculty of Medicine, KU Leuven, Leuven, Belgium; ^4^ CP Reference Center, University Hospitals Leuven, Leuven, Belgium; ^5^ Department of Development and Regeneration, KU Leuven, Leuven, Belgium; ^6^ Pediatric Orthopedics, Department of Orthopedics, University Hospitals Leuven, Leuven, Belgium; ^7^ Biostatistics Unit, Department of Public Health and Primary Care, Ghent University, Ghent, Belgium; ^8^ Clinical Motion Analysis Laboratory, University Hospitals Leuven, Pellenberg, Belgium

**Keywords:** spastic cerebral palsy, progressive resistance training, muscle morphology, lower extremity, isometric muscle strength, functional muscle strength, ultrasonography

## Abstract

Children with spastic cerebral palsy often present with muscle weakness, resulting from neural impairments and muscular alterations. While progressive resistance training (PRT) improves muscle weakness, the effects on muscle morphology remain inconclusive. This investigation evaluated the effects of a PRT program on lower limb muscle strength, morphology and gross motor function. Forty-nine children with spastic cerebral palsy were randomized by minimization. The intervention group (nparticipants = 26, age: 8.3 ± 2.0 years, Gross Motor Function Classification System [GMFCS] level I/II/III: 17/5/4, nlegs = 41) received a 12-week PRT program, consisting of 3-4 sessions per week, with exercises performed in 3 sets of 10 repetitions, aiming at 60%–80% of the 1-repetition maximum. Training sessions were performed under supervision with the physiotherapist and at home. The control group (nparticipants = 22, age: 8.5 ± 2.1 year, GMFCS level I/II/III: 14/5/3, nlegs = 36) continued usual care including regular physiotherapy and use of orthotics. We assessed pre- and post-training knee extension, knee flexion and plantar flexion isometric strength, rectus femoris, semitendinosus and medial gastrocnemius muscle morphology, as well as functional strength, gross motor function and walking capacity. Data processing was performed blinded. Linear mixed models were applied to evaluate the difference in evolution over time between the control and intervention group (interaction-effect) and within each group (time-effect). The α-level was set at *p* = 0.01. Knee flexion strength and unilateral heel raises showed a significant interaction-effect (*p* ≤ 0.008), with improvements in the intervention group (*p* ≤ 0.001). Moreover, significant time-effects were seen for knee extension and plantar flexion isometric strength, rectus femoris and medial gastrocnemius MV, sit-to-stand and lateral step-up in the intervention group (*p* ≤ 0.004). Echo-intensity, muscle lengths and gross motor function showed limited to no changes. PRT improved strength and MV in the intervention group, whereby strength parameters significantly or close to significantly differed from the control group. Although, relative improvements in strength were larger than improvements in MV, important effects were seen on the maintenance of muscle size relative to skeletal growth. In conclusion, this study proved the effectiveness of a home-based, physiotherapy supervised, PRT program to improve isometric and functional muscle strength in children with SCP without negative effects on muscle properties or any serious adverse events.

**Clinical Trial Registration**: ClinicalTrials.gov, identifier NCT03863197.

## 1 Introduction

Cerebral palsy (CP) is one of the main causes of childhood-onset physical disability (Rosenbaum et al., 2007). When classified according to neuromuscular presentation, spastic CP (SCP) is the largest subcategory, whereas a topographical classification further divides SCP in unilateral and bilateral involvement (Surveillance of Cerebral Palsy in Europe, 2002). CP is caused by an injury in the developing brain and the primary neuromotor symptoms are related to upper motor neuron damage, including muscle weakness, spasticity, reduced selective motor control (SMC), and a lack of balance and coordination (Graham et al., 2016). These neuromotor symptoms can lead to secondary musculoskeletal symptoms like reduced joint range of motion, altered muscle growth and bony deformities, which may further influence muscle weakness (Graham et al., 2016). Altogether, these neuromotor symptoms lead to limitations in activities and participation (Rosenbaum et al., 2007).

The initial brain injury in children with SCP leads to an altered neural drive to skeletal muscles and subsequent alterations in movement and muscle activation patterns (Clowry, 2007; Gough and Shortland, 2012). Consequently, skeletal muscle tissue of children with SCP shows a lack of both longitudinal and cross-sectional fiber growth starting in the postnatal period and evolving into childhood (Handsfield et al., 2016; Herskind et al., 2016; Willerslev-Olsen et al., 2018). Decreased muscle size is reported both in proxy measures of fiber width like anatomical CSA, muscle thickness and muscle volume (MV) (Moreau et al., 2010; Noble et al., 2014b; Handsfield et al., 2016; Schless et al., 2017; Hanssen et al., 2021), as well as in proxy measures of fiber length like muscle length (ML) and fascicle length (Matthiasdottir et al., 2014; Schless et al., 2017; Hanssen et al., 2021). Additionally, the quality of muscle tissue is reduced due to increases in fatty and fibrillar tissue in the muscle, resulting in an increased proportion of the MV comprising non-contractile material (Noble et al., 2014a; Pitcher et al., 2015; Obst et al., 2017; Schless et al., 2019).

Muscle morphology has been considered as the primary determinant of force production and the combination of reduced muscle size and quality leads to muscle weakness in children with SCP (Lieber and Friden, 2000). However, while general decreases in muscle size of 18%–50% have been described in ambulant children with SCP (Moreau et al., 2010; Noble et al., 2014b; Handsfield et al., 2016; Schless et al., 2017; Massaad et al., 2019; Hanssen et al., 2021), muscle weakness was found to be more profoundly present, specifically in lower limb muscles, with deficits up to 80% (Wiley and Damiano, 1998; Goudriaan et al., 2018; Darras et al., 2021; Hanssen et al., 2021). We recently showed that the contribution of this decreased muscle size to muscle weakness was 23%–57% for muscle groups around the knee and ankle joint, however, with high variability between children (Hanssen et al., 2021). The remaining part of muscle weakness may result from other muscular changes not directly reflected in muscle size, such as altered fascicle length and pennation angle, as well as from the neural ability to fully recruit motor units and selectively activate the muscle. In children with SCP, the latter presents as an inability to maximally activate agonists and an increased antagonist co-contraction (Elder et al., 2003; Mockford and Caulton, 2010).

Since skeletal muscle tissue is adaptive to mechanical load, one of the best treatment options for muscle weakness is progressive resistance training (PRT) (Faigenbaum and Myer, 2010). Initial strength improvements through PRT result from neural adaptations (Sale, 1988; Tesch, 1988), but PRT can eventually elicit significant adaptations in muscle mass in healthy adults, also referred to as muscle hypertrophy (Franchi et al., 2014; Schoenfeld et al., 2017a). Training results are influenced by the manipulation of training variables (American College of Sports Medicine, 2009), and the recommendations for pediatric training variables focusing on muscle strength include a frequency of 2–3 times per week, with 1-3 sets, using loads that correspond to a repetition range of a 6–15 repetition maximum with qualified instruction and close supervision (Faigenbaum and Myer, 2010; Lloyd et al., 2014).

The effects of PRT have been extensively studied in the SCP population. Recent reviews indicated positive effects of PRT on muscle weakness, however the results on gross motor function and gait speed remain inconclusive (Ryan et al., 2017; Liang et al., 2021; Merino-Andrés et al., 2021). Moreover, the quality of evidence is often rated as low to very low (Ryan et al., 2017). The studies evaluating the underlying mechanisms to increased muscle strength, focusing on muscle morphology, are rather limited. A review by Gillett et al. (2016) found 6 studies showing preliminary evidence that PRT leads to muscle hypertrophy in children and adolescents with CP (Gillett et al., 2016). However, two recent investigations indicated limited to no effects on muscle size outcomes after PRT in children and adolescents with CP (Kruse et al., 2019; Ryan et al., 2020) and an additional review also reported inconsistent evidence with only low-to-moderate quality investigations (Walhain et al., 2020). On the other hand, in young adults, aged 15–30 years, significant increases in plantar flexor MV have been found after 12 weeks of combined anaerobic and strength training (Gillett et al., 2018), and Cho and Lee (2020) reported an increased rectus femoris CSA and quadriceps thickness in young children with SCP after 6 weeks of functional PRT. It remains challenging to generalize these findings, due to the lack of robust designs and variation in included age range, the type of training used, the duration of the training program, the muscle group(s) trained, the assessments used for muscle strength and size, and the training location (Gillett et al., 2016; Walhain et al., 2020; Moreau and Lieber, 2022).

Previous investigations used prospective cohort studies or randomized controlled trials (RCTs) comparing 2 different exercise intervention programs with each other, without a usual care control group (Stackhouse et al., 2007; McNee et al., 2009; Moreau et al., 2013; Williams et al., 2013; Lee et al., 2015; Kruse et al., 2019). So far, 3 RCTs have been performed comparing the effects of PRT with a usual care control group assessed at identical time points, 2 in adolescents and young adults focusing on the plantar flexors (Gillett et al., 2018; Ryan et al., 2020) and 1 in children focusing solely on the knee extensors (Cho and Lee, 2020). One of these RCTs in adolescents found no effects of PRT, in contrast to the other 2 (Ryan et al., 2020). Assessing the effects of PRT on muscle strength, size and function, next to a usual care control group, for several muscle groups, will improve our understanding of the adaptive potential of skeletal muscle in children with SCP and the mechanisms underpinning strength or functional improvements.

Therefore, the primary goal of this investigation was to evaluate the effects of a 12-week PRT program on muscle strength and muscle size of the lower limbs in children with SCP, in comparison to a usual care control group. Secondary, the effects on muscle length and quality, as well as on gross motor function and walking capacity, were assessed. We hypothesized an increase in isometric strength and MV in the intervention group compared to the control group, with unchanged intrinsic muscle quality in both groups. With part of the strength increases resulting from neural adaptations, the increases in muscle strength were expected to be larger than the increases in muscle size. We further expected ML to increase similarly in both groups due to general growth, and improved functional strength in the intervention group, whilst gross motor function and walking capacity were expected to remain unchanged.

## 2 Materials and methods

A waitlist RCT was conducted to define the effects of a PRT program for the knee extensor, knee flexor and plantar flexor muscle groups. A minimization technique was used to divide participants in an intervention group, performing a 12-week PRT program and a waitlist control group, continuing usual care for 12 weeks first. The data of the waitlist control group were only included as control data, the results of the delayed intervention have not been included because this was out of the scope of the current investigation.

### 2.1 Participants

Participants were recruited across Flanders in Belgium, through the CP reference center of the University Hospitals Leuven, pediatric physiotherapists, and special needs schools between August 2018 and February 2021. Written informed consent was obtained from the children’s parents or legal guardian prior to the first assessment. The study was registered at Clinicaltrials.gov (NCT03863197) and was approved by local medical ethical committees of University Hospitals Leuven (s59945) and Ghent (EC/2017/0526). The inclusion criteria for participation were 1) a diagnosis of SCP, 2) age at baseline between 5 and 11 years and 3) level of I, II or III on the gross motor function classification system (GMFCS) (Palisano et al., 2008). Exclusion criteria were 1) botulinum neuro-toxin A injections and/or lower leg casts in the past 6 months, 2) lower limb bony surgery in the past 2 years, 3) lower limb muscular surgery or selective dorsal rhizotomy at any time point, 4) inability to communicate in Dutch or English and 5) inability to understand instructions and cooperate during assessments and training.

### 2.2 Study design

After enrollment and prior to the first assessment, participants were assigned to the intervention or the control group through randomization by minimization. MinimPy was used to randomize the participants based on age (2 levels: <8 years and >8 years) and GMFCS score (3 levels: I, II and III) (Saghaei and Saghaei, 2011). The allocation ratio was 1:1 and probability was set at 0.75. The biased coin minimization was used as probability method and marginal balance as distance measure. The global Covid-19 pandemic led to a forced stop of the investigation in March 2020, with recommencement after several months. Since drop-outs could not be administered in MinimPy, group assignment after recommencement was performed manually by an independent researcher to prevent skewness in the balance of the 2 groups, whereby the same minimization procedure, based on age and GMFCS level, was applied.

Assessments were planned in the University Hospitals and special needs schools, at baseline (PRE) and after the 12-week control or intervention period (POST). Training and assessments were always conducted on different days. Participants in the intervention group started training within 2 weeks after the baseline assessment. For both groups, the POST assessments were performed within 1 week after the 12-week period. Blinding was not completely possible since participants and trainers were aware of group allocation. Assessors were aware of group allocation during assessments but data was anonymized before processing and analyses. The participants were PRE and POST assessed by the same assessors and PRE and POST data was processed by the same processer.

Before the start of the trial, piloting for the feasibility of the measurements and parameter extraction was done through prior and parallel investigations. The exercises used during the training were piloted through iterations with strength and conditioning specialists to ensure correct exercise selection and also with senior pediatric physiotherapists and their patients to ensure the applicability in the targeted population of children with SCP.

### 2.3 Sample size

At trial commencement in 2018, no data were available on an RCT investigating changes in muscle size after PRT in a population of children with SCP. Therefore, effect sizes (d) of systematic reviews and meta-analyses reporting muscle strength outcomes after PRT were used. Reported effect sizes were moderate (d = 0.53) (Ryan et al., 2017) and large (d = 1.050 and d = 1.105) (Park and Kim, 2014). Based on a large effect size of d = 0.8 with the probability of making a type I error set equal to or less than 1% (*p* ≤ 0.01) and a power of 80% an independent samples t-test with common standard deviation required 39 independent observations per group to compare the evolution over time between the intervention and control groups. To account for the possibility of clustered legs in bilaterally affected participants a design effect (Killip et al., 2004) was calculated resulting in an estimated sample size of clustered observations per group with following formulas:

Design effect = 1 + ρ ∗(m−1) 

where *ρ* is the intraclass correlation and *m* the cluster size, and,

Estimatedsamplesizeofclusteredlegs=estimatedsamplesizeofindependentobservations*designeffect=m*numberofparticipants.

With a cluster size of m = 2 (2 legs per bilaterally affected participant) and an intraclass correlation coefficient of *ρ* = 0.1, based on literature of general health parameters (Adams et al., 2004), the estimated design effect was 1.1, resulting in 43 clustered legs, or 22 bilaterally affected participants per group. A group including both uni- and bilaterally affected children would require a sample size between 39 and 43.

### 2.4 Intervention

Participants assigned to the control group continued their usual care. Participants assigned to the intervention group undertook 3 or 4 PRT sessions per week (alternating), on non-consecutive days, for 12 weeks, resulting in a total of 42 scheduled sessions. The PRT program was explained during a visit to the physiotherapist(s) and parent(s). After a familiarization phase of up to 3 sessions, the official PRT started. At least 1, but up to 3, sessions per week were performed under the supervision of the physiotherapist(s). The remaining sessions were completed at home under the supervision of a parent or guardian. The patient-specific PRT program was created and communicated through the online software platform Skill-Up (www.Skill-up.com) and each PRT session was logged in a training diary. Throughout the 12-week period, the PRT program was supervised through phone calls, e-mails, and targeted visits to check adherence and progress exercises by trainers with a background in human movement sciences (BH and NP). Participants in the intervention group were invited for an interim assessment after 6 weeks, which was also used as an additional moment to evaluate and adjust the intensity of the training program. The results of this interim assessment are not included in the current study.

The PRT program, targeting strength and hypertrophy of knee extensors, knee flexors and plantar flexors, was prescribed in close consultation with the personal physiotherapist(s) to ensure feasibility for each participant. It was recommended to begin the sessions with a 5-min dynamic warm-up and end with a 5-min cool-down. Depending on the participants’ abilities, the PRT started with 1-3 functional multi-joint exercises, followed by 2-3 single-joint exercises targeting the specific muscle groups, all performed without orthotics. Examples of used exercises are presented in Supplementary Table S1. Following international guidelines for youth resistance training of the National Strength and Conditioning Association, as well as CP-specific recommendations, the initial exercises started at a training volume of 3 sets of 10 repetitions, aiming at an exercise intensity of 60%–80% of the estimated 1-repetition maximum, with a rest period in between sets of at least 1 min (Faigenbaum et al., 2009; Verschuren et al., 2011; Lloyd et al., 2014; Verschuren et al., 2016). The most difficult exercise that could be performed, with the maximal load that could be lifted, for the defined number of repetitions and sets was defined through trial and error (Moreau, 2020). Throughout the training program, exercise difficulty was increased. Thereto, physiotherapists were encouraged to regularly test the difficulty of the exercises, i.e., at least biweekly, by asking a maximum performance in the last set, based on exercise execution and fatigue. The intensity was modified by the trainers or the physiotherapist(s) if the last set exceeded 15 well-executed repetitions. Exercise modification options were, depending on the exercise, addition of weight in a weighted vest (0.5 kg increments up to 10 kg), use of a stronger resistance band (5 colors ranging from light to very heavy), increase of ankle weight (1.0, 1.5, or 2.0 kg), or selection of a more challenging exercise. The number of sets and repetitions performed, modifications or compensations, perceived fatigue at the end of the session and potential adverse events were noted in the training diary.

### 2.5 Assessments and outcome measures

#### 2.5.1 Participant characteristics

Age, body mass and height were recorded for the participants at the PRE- and POST-assessment. Usual care and treatment history were requested at the PRE-assessment using standardized in-house questionnaires. A routine clinical examination regarding the investigated muscle groups was performed, including passive joint range of motion, spasticity, manual muscle testing and SMC, based on standard clinical scales (Matthews, 1977; Bohannon and Smith, 1987; Boyd, 1999; Gage et al., 2009). These clinical examination parameters were used to define the most affected leg for bilaterally affected children. If both sides were equally affected, the right leg was used. Lower limb SMC was assessed with the selective control assessment of the lower extremity total limb score (Fowler et al., 2009). Assessments were commonly performed in the same order, starting with clinical examination and muscle morphology, followed by isometric and functional strength and lastly gross motor function and walking capacity. In some exceptions, such as time and space limitations when assessing in schools, this order could be modified. An overview of the clinimetric properties of each assessment can be found in Supplementary Table S2.

#### 2.5.2 Isometric strength assessments

Knee extensor, knee flexor and plantar flexor maximum voluntary isometric contractions were collected with a fixed dynamometer in a previously described, custom-designed chair (Goudriaan et al., 2018; Verreydt et al., 2022). The dynamometer was positioned at 75% of the lower leg and foot length, and the lever arm distance was measured between this position and the joint axis. After a test trial, the aim was to collect 3 well executed maximum voluntary isometric contractions with a duration of 3–5 s. Standardized verbal encouragement and visual feedback were provided throughout the isometric contraction. The protocol included periods of rests of at least 10 s between repeated assessments and of at least 2 min between different muscle groups. Peak force was extracted from each trial with a custom-written MATLAB script and maximal joint torque (in Nm) was calculated by multiplying peak force with the lever arm and taking the average of the 3 trials. Knee and plantar flexion maximal voluntary isometric contractions were corrected for gravity by subtracting the gravitational torque in rest from the outcomes. Maximal joint torque was divided by body weight to obtain normalized maximal joint torque (Nm/kg).

#### 2.5.3 Three-dimensional freehand ultrasonography

A previously described (Hanssen et al., 2021) and validated (Cenni et al., 2016) 3-dimensional (3-D) freehand ultrasonography technique was used to evaluate muscle morphology, combining a 2-dimensional B-mode ultrasonography device (Telemed-Echoblaster 128 Ext-1Z, with a 5.9 cm 10 MHz linear ultrasound transducer, Telemed, Ltd., Lithuania) with a motion tracking system (Optitrack V120:Trio, NaturalPoint, Inc., Corvallis, Oregon, United States). Ultrasound settings were kept constant throughout the study period at a frequency of 8 MHz, with a focus of 3 cm, a gain of 64%, a dynamic range of 56 dB and unaltered time-gain compensation. Depth could vary between 5 and 7 cm, adjusted to muscle size. The m. rectus femoris was scanned in supine position with a triangular cushion underneath the calf, providing approximately 25 degrees of knee and hip flexion. The m. semitendinosus and m. medial gastrocnemius were scanned in prone position with a triangular cushion underneath the shank, providing 25 degrees of knee flexion and an unconstrained ankle angle. STRADWIN software (version 6.0; Mechanical Engineering, Cambridge University, Cambridge, United Kingdom) was used for data acquisition, generation of 3-D datasets, and data processing. Experienced processers drew equally spaced transverse plane segmentations throughout the 3-D datasets, which were interpolated with an automatic cubic planimetry technique resulting in the MV (in mL) (Treece et al., 1999). ML (in mm) was defined by calculating the linear distance between muscle origin and muscle tendon junction. Both MV and ML were normalized to fibula length to correct for skeletal growth (mL/cm and cm/cm). The echo-intensity (EI) from ultrasound images was used as an indication of intrinsic muscle quality, with higher EI-values representing an increased ratio of non-contractile vs. contractile tissue (Pitcher et al., 2015; Young et al., 2015). EI (expressed in arbitrary units) was computed as the average value throughout the interpolated reconstruction of the muscle.

#### 2.5.4 Functional strength assessments

Endurance functional strength was assessed with 30-s maximum repetition tests including bi- and unilateral heel raise, sit-to-stand, and lateral step-up, as previously described (Van Tittelboom et al., 2021). Participants completed as many repetitions as possible in 30 s. Each test was repeated 2 to 3 times and the average was taken as final score. Explosive strength was evaluated with a standing long jump, with both feet together, which was also repeated 3 times and the scores were averaged. The use of orthoses was not allowed, as the PRT program was also performed without orthoses.

#### 2.5.5 Gross motor function and walking capacity

Gross motor function was evaluated with the Gross Motor Function Measure-Item Set (Brunton and Bartlett, 2011) and the Gross Motor Ability Estimator 2 was used to estimate the final score. The 1-min walk test, performed on an indoor 20-m track, was used to assess walking capacity (McDowell et al., 2009; Chrysagis et al., 2014). The participant was instructed to walk as fast as possible without running and the distance covered in 1 min was recorded. Both assessments were performed without orthoses, but a kay-walker was allowed for the 1-min walk test for children classified as GMFCS-level III.

### 2.6 Statistics

Descriptive statistics for each group were summarized using means and standard deviations. Linear mixed models were applied to evaluate the difference in evolution over time between the control and intervention group (time*group interaction-effect) and between PRE and POST within each group (time-effect). The random effects in these models can correct for the correlation between repeated observations within the same subject. Moreover, in the case of missing values that are completely at random or at random, linear mixed models provide valid inferences for missing observations. A covariance structure for repeated measurements to model longitudinal dependencies within the participant was applied with a random intercept for participant (legs are nested within participants). A full-factorial time*group mean model adjusted for age and GMFCS level, with a compound symmetry covariance matrix was used, unless an unstructured covariance matrix resulted in a better fit (Akaike information criterion decrease of >2). Adjustment for age (categorical variable: <8 years and >8 years) and GMFCS level was included as these were used to allocate the children to the control or intervention group. The primary analyses were performed on all affected legs of all participants (both legs of bilateral affected children and the affected leg of unilateral affected children), also including available data of participants who dropped out. To determine if the inclusion of all affected legs, the change in randomization from program-driven to manual and the participants who dropped out influenced the results, sensitivity analyses were performed on the most affected legs of the participants that were originally randomized before onset of the pandemic as well as on the most affected legs of the participants who participated in the POST assessments after the intervention or control period. The α-level was adjusted to *p* ≤ 0.01 since multiple parameters were evaluated for most research questions, with a maximum of 5 parameters (functional strength). All analyses were performed with SPSS (Version 28, SPSS Inc., Chicago, Illinois). The mean difference scores were compared with the known standard error of measurements and minimal detectable changes as presented in Supplementary Table S2. Presented relative changes indicate the mean difference as a percentage of the baseline values. There were some missing data due to 3DfUS reconstructions that could not be (fully) analyzed because of technical errors, isometric and functional strength tests that could not be assessed due to inability of the participant and missing anthropometric information.

## 3 Results

### 3.1 Participants

**FIGURE 1:**
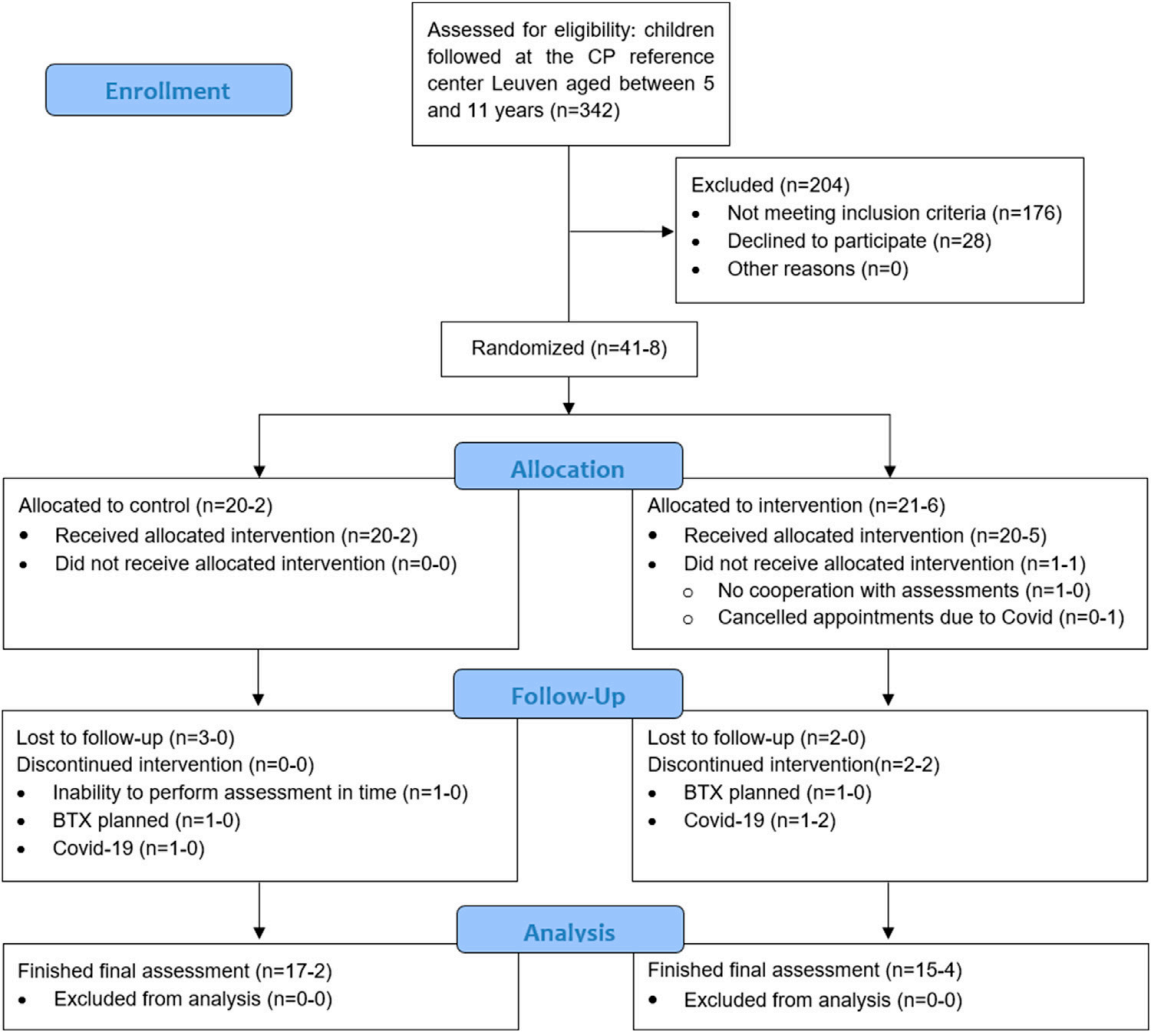
All children aged 5–11 years old were extracted from the clinical database of the CP reference center Leuven (*n* = 342) and screened based on gross motor function classification system level and type of CP. The scheduled appointments at the children’s hospital and gait laboratory were monthly checked to further screen potential participants based on the exclusion criteria (±10% eligible every month, part of the *n* = 342). Additionally, pediatric physiotherapists at private practices and special needs schools were consulted for potential participants resulting in 6 of the 49 participants not being followed up in Leuven. The first number, before the hyphen, represents the participants that were randomized by minimization by Minim-Py before the forced stop of the study due to the global Covid-19 pandemic, the number after the hyphen represents the participants who were manually minimized afterwards. *Included in primary analyses: control group=22 and intervention group=26 (1 randomized child did not participate in baseline assessments). Included in sensitivity analyses: • Randomized: control group=20 and intervention group=20 (1 randomized child did not participate in baseline assessments). • Finished: control group=19 and intervention group = 19. BTX, botulinum neurotoxin type A; CP, cerebral palsy.

The participant flow chart for recruitment, allocation and drop-out, following CONSORT guidelines, is presented in Figure 1. Eventually, 49 children participated in this investigation, of whom 41 were randomized by minimization in the MinimPy software and 8 were added after the enforced stop due to Covid-19 pandemic in March 2020. The main reasons for refusal to participate in the study were time and distance constraints or the child being in a challenging period regarding school or home situation. Initially, 20 children were allocated to the control group, of whom 17 completed the POST-assessments. Twenty-one children were allocated to the intervention group, and 15 completed the intervention and participated in the POST-assessments. Of the 8 additional children included post Covid-19 regulations, 2 were manually allocated to the control group and 6 to the intervention group, with a drop-out of 2 children in the latter. Drop-outs in the control group were due to: 1) inability to perform the POST-assessments in time due to medical reasons (*n* = 1); 2) a clinically prescribed botulinum neurotoxin-A injection during the control period (*n* = 1) and 3) Covid-19 regulations (*n* = 1). Drop-outs in the intervention group were due to 1) a lack of cooperation during the assessments (*n* = 1); 2) cancellation of appointments due to Covid, eventually leading to loss of contact (*n* = 1); 3) a clinically prescribed botulinum neurotoxin-A injection during the intervention period (*n* = 1); 4) Covid-19 regulations (*n* = 3).

Patient characteristics for the primary analyses on all affected legs of all participants in both groups are presented in Table 1, and for both sensitivity analyses in Supplementary Table S3. At baseline the control group (*n* = 22) had an average age 8.5 ± 2.1 years, with a weight of 28.3 ± 7.1 kg and a height of 128.6 ± 11.2 cm. In this group 14 children were classified as GMFCS level I, 5 as GMFCS level II and 3 as GMFCS level 3. Eight children were unilaterally affected and 14 bilaterally, resulting in 36 assessed legs. Average fibula length of all assessed legs was 27.6 ± 3.5 cm and selective control assessment of the lower limb score was 7.1 ± 2.6. For the intervention group (*n* = 26) the average age was 8.3 ± 2.0 years, with an average weight of 27.7 ± 8.1 kg and 127.3 ± 14.0 cm. Seventeen children were classified as GMFCS level I, 5 as GMFCS level II and 4 as GMFCS level 3. Eleven children were unilaterally affected and 15 bilaterally, resulting in 41 assessed legs. Fibula length was on average 27.3 ± 3.6 cm and the selective control assessment of the lower limb score was 7.0 ± 2.1. One participant was randomized but never assessed due to repeated cancellations, therefore the total number of participants in the primary analyses is 48 (n_participants_ = 48, n_affected legs_ = 77).

**TABLE 1:** Descriptive statistics of patient characteristics for all participants (or legs) included in the primary analyses.

	**CON (n=22)**	**INT (n=26)**
	**Frequencies (%)**	**Frequencies (%)**
GMFCS		
Level I	14 (63.6)	17 (65.4)
Level II	5 (22.7)	5 (19.2)
Level III	3 (13.6)	4 (15.4)
Age group		
<8 years	11 (50.0)	12 (46.2)
>8 years	11 (50.0)	14 (53.8)
Involvement		
Unilateral	8 (36.4)	11 (42.3)
Bilateral	14 (63.6)	15 (57.7)
Gender		
Boy	16 (72.7)	14 (53.8)
Girl	6 (27.3)	12 (46.2)

	Mean ± SD	Mean ± SD
Age (years)	8.5 ± 2.1	8.3 ± 2.0
Weight (kg)	28.3 ± 7.1	27.7 ± 8.1
Height (cm)	128.6 ± 11.2	127.3 ± 14.0

	CON (n=36)	INT (n=41)
	Mean ± SD	Mean ± SD
Fibula (mm)	27.6 ± 3.5	27.3 ± 3.6
SCALE	7.1 ± 2.6	7.0 ± 2.1

Total participants in control group = 22 and in intervention group = 26. Total legs in control group = 36 and in intervention group = 41.

Abbreviations: CON, control group; GMFCS, gross motor functional classification system; INT, intervention group; N, number; SCALE, selective control assessment of the lower extremity; SD, standard deviation.

Units: cm, centimeter; kg, kilogram; mm, millimeter.

### 3.2 Training program and usual care

The median number of performed training sessions was 35 (83% adherence), ranging from 24 to 42 (57%–100% adherence). The PRT part of the training sessions, without warm-up and cool-down, lasted between 25 and 45 min. Fatigue scores at the end of each training session showed a wide variability between and within participants, ranging from 0 to 10, with a median of 3 out of 10. However, physiotherapists and parents often mentioned a discrepancy between reported fatigue by the child and perceived fatigue based on exercise performance. Reported adverse events were muscle cramp or pain, joint pain and general discomfort from the weighted vest. No adverse events were reported that led to missed training sessions or required medical treatment. In case of pain during or shortly after the exercises, individual exercise intensity was modified by reducing resistance or changing the exercise, followed by exercise progression once pain had disappeared.

Usual care consisted of the use of orthoses and regular physical therapy including, among others, stretching, muscle strengthening and gait training. Regular physical therapy ranged from 1 to 4 sessions per week. Although usual care involved general strength exercises, no physiotherapist reported PRT as part of usual care, whereby PRT was defined as performing several sets of each exercise with gradually increasing load.

### 3.3 Primary analyses

**FIGURE 2:**
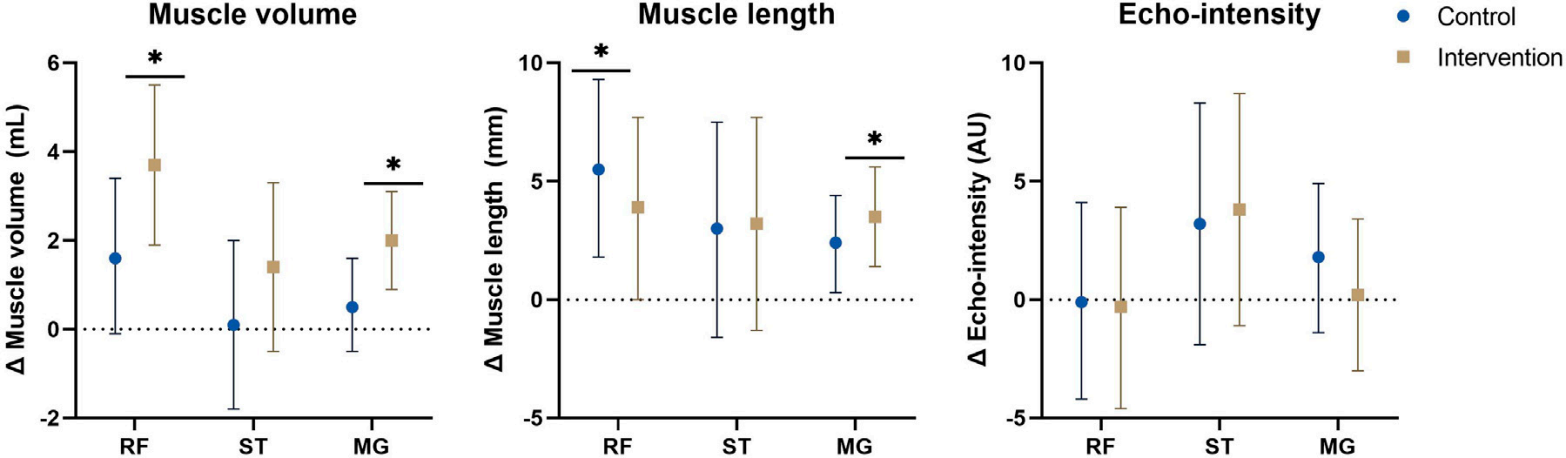
Estimated marginal mean differences with 95% confidence interval of mixed model analyses for muscle morphology with results for within and between analyses including all participants and all affected legs. *: significant time-effect at *p* ≤ 0.01. Abbreviations: Δ, change; MG, medial gastrocnemius; RF, rectus femoris; ST, semitendinosus. Units: AU, arbitrary units; mL, milliliter; mm, millimeter.

**FIGURE 3:**
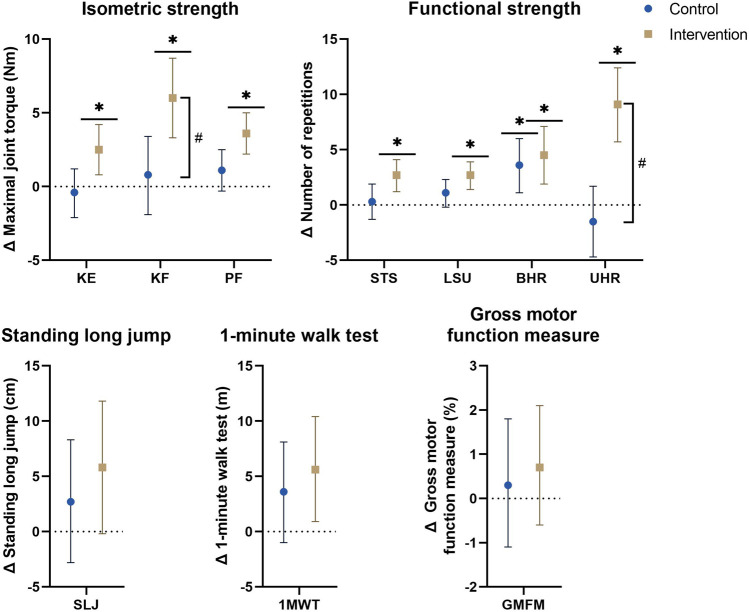
Estimated marginal mean differences with 95% confidence interval of mixed model analyses for strength and functional parameters with results for within and between analyses including all participants and all affected legs for bilaterally tested parameters. #: significant time*group interaction-effect at *p* ≤ 0.01. *: significant time-effect at *p* ≤ 0.01. Abbreviations: Δ, hange; 1MWT, 1-minute walk test; BHR, bilateral heel raise; GMFM, gross motor function measure; KE, knee extension; KF, knee flexion; LSU, lateral step-up; PF, plantar flexion; SLJ, standing long jump; STS, sit-to-stand; UHR, unilateral heel raise. Units: Cm, centimeter; M, meter; Nm, Newton-meter.

Baseline data for the primary analyses on all affected legs of all participants in both groups are presented in Table 2. The mean differences of the mixed model analyses are presented in Figure 2 for the morphological parameters and in Figure 3 for the strength and functional parameters, supported by the estimated marginal means of the PRE- and POST-assessments in Supplementary Tables S5, S6. Isometric muscle strength showed a significant interaction-effect for knee flexion (*p* = 0.008), whilst knee extension and plantar flexion were close to significance (*p* ≤ 0.015). In the intervention group, all muscle groups showed a significant time-effect, indicating an increase in isometric muscle strength (knee flexion: Δ = 6.0Nm (3.3–8.7), p=<0.001, knee extension: Δ = 2.5Nm (0.8–4.2), *p* = 0.004, plantar flexion: Δ = 3.6Nm (2.2–5.0), *p* < 0.001). The results for normalized isometric muscle strength were similar, with a significant interaction-effect for knee flexion (*p* = 0.005) and significant time-effects in the intervention group for knee flexion (Δ = 0.19Nm/kg (0.11–0.27), *p* < 0.001) and plantar flexion (Δ = 0.13Nm/kg (0.08–0.18), *p* < 0.001) whilst knee extension was close to being significant (Δ = 0.07Nm/kg (0.01–0.13), *p* = 0.032).

**TABLE 2:** Descriptive statistics of observed data at baseline for all participants, with all affected sides for unilaterally tested parameters.

			CON		INT
		n	Mean ± SD	n	Mean ± SD
Isometric strength	KE (Nm)	36	16.6 ± 11.1	39	14.3 ± 9.5
	KF (Nm)	36	13.7 ± 11.3	39	8.1 ± 8.2
	PF (Nm)	35	7.9 ± 5.9	39	5.6 ± 3.7
Muscle volume	RF (mL)	35	70.5 ± 24.1	41	63.8 ± 27.0
	ST (mL)	31	48.9 ± 16.5	38	48.3 ± 18.4
	MG (mL)	35	48.2 ± 28.3	41	42.1 ± 19.0
Muscle length	RF (mm)	33	225.6 ± 31.5	40	222.3 ± 34.9
	ST (mm)	31	217.0 ± 34.6	35	211.6 ± 27.0
	MG (mm)	34	154.4 ± 31.6	41	150.8 ± 24.3
Muscle quality	RF (AU)	35	140.8 ± 15.4	41	140.5 ± 17.7
	ST (AU)	31	138.7 ± 16.0	38	134.4 ± 19.3
	MG (AU)	35	161.3 ± 12.5	41	162.5 ± 14.0
Functional strength	STS (n)	21	13.0 ± 5.1	25	14.4 ± 4.9
	LSU (n)	34	17.1 ± 8.7	38	16.0 ± 6.5
	BHR (n)	22	23.8 ± 10.4	22	24.0 ± 9.3
	UHR (n)	27	20.7 ± 10.8	27	15.1 ± 9.4
	SLJ (cm)	16	84.1 ± 30.1	19	75.9 ± 28.3
Walking capacity	1MWT (m)	21	77.6 ± 24.2	22	76.9 ± 20.5
Gross motor function	GMFM (%)	18	81.0 ± 12.6	25	79.6 ± 14.5

Complete dataset: participants in control group = 22 and in intervention group = 26. Total legs in control group = 36 and in intervention group = 41.

Abbreviations: 1MWT, 1-min walk test; BHR, bilateral heel raise; CON, control group; GMFM, gross motor function measure; INT, intervention group; KE, knee extension; KF, knee flexion; LSU, lateral step-up; MG, medial gastrocnemius; PF, plantar flexion; RF, rectus femoris; SD, standard deviation; SLJ, standing long jump; ST, semitendinosus; STS, sit to stand; UHR, unilateral heel raise.

Units: AU, arbitrary units on 8-bit greyscale; cm, centimeter; m, meter; mL, milliliter; mm, millimeter; n, number; Nm, Newton meter.

For MV, no significant interaction-effects were present, however, the increase in rectus femoris and medial gastrocnemius MV showed a significant time effect in the intervention group (rectus femoris: Δ = 3.7 ml (1.9–5.5), *p* < 0.001, medial gastrocnemius: Δ = 2.0 ml (0.9–3.1), *p* < 0.001). In the control group, the increase of rectus femoris MV was close to significant (Δ = 1.6 ml (−0.1–3.4), *p* = 0.069). No interaction-effects were found for ML and EI. In both groups a significant or close to significant time-effect was found for rectus femoris ML (CON: Δ = 5.5 mm (1.8–9.3), *p* = 0.005, INT: Δ = 3.9 mm (0.0–7.7), *p* = 0.050) and medial gastrocnemius ML (CON: Δ = 2.4 mm (0.3–4.4), *p* = 0.025, INT: Δ = 3.5 mm (1.4–5.6), *p* = 0.002), indicating increased ML. For ML of the semitendinosus and EI of all 3 muscles, no significant time-effects were found.

**FIGURE 4:**
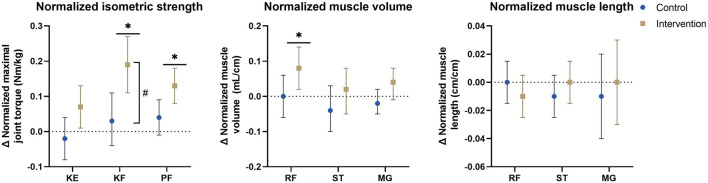
Estimated marginal mean differences with 95% confidence interval of mixed model analyses for normalized muscle morphology and isometric strength parameters with results for within and between analyses including all participants and all affected legs. #: significant time*group interaction-effect at *p* ≤ 0.01. *: significant time-effect at *p* ≤ 0.01. Abbreviations: Δ, change; KE, knee extension; KF, knee flexion; MG, medial gastrocnemius; PF, plantar flexion; RF, rectus femoris; ST, semitendinosus. Units: Cm/cm, centimeter/centimeter; mL/cm, milliliter per centimeter; Nm/kg, Newton-meter per kilogram.

Regarding normalized morphological parameters, mean differences of the mixed model analyses are presented in Figure 4 and estimated marginal means of the PRE- and POST-assessment are included in Supplementary Table S7, showing close to significant interaction-effects were present for normalized MV of the rectus femoris (*p* = 0.041) and medial gastrocnemius (0.074), With a significant or close to significant time-effect in the intervention group (rectus femoris: Δ = 0.08 ml/cm, (0.02–0.14), *p* = 0.007), medial gastrocnemius: Δ = 0.04 ml/cm, (−0.01–0.08), *p* = 0.085) and absent time-effects in the control group (rectus femoris: Δ = 0.00 ml/cm, (−0.06–0.06), *p* = 0.941), medial gastrocnemius: Δ = −0.02 ml/cm, (-0.05–0.02), *p* = 0.450). For normalized ML no interaction- nor time-effects were found, with mean differences in both groups around 0 cm/cm.

The endurance functional strength assessments indicated significant interaction-effects for unilateral heel raise (*p* < 0.001), and was close to significance for sit-to-stand and lateral step-up (*p* ≤ 0.076), all with significant time-effects in the intervention group indicating an increase in number of repetitions (unilateral heel raise: Δ = 9.1 (5.7–12.4), *p* < 0.001, sit-to-stand: Δ = 2.7 (1.2–4.1), *p* < 0.001, lateral step-up: Δ = 2.7 (1.4–3.9), *p* < 0.001). The bilateral heel raise improved significantly in both groups [CON: 3.6 (1.1–6.0), *p* = 0.005, INT: 4.5 (1.9–7.1), *p* = 0.001]. Explosive functional strength, walking capacity and gross motor function showed no significant interaction-effects, but the time-effects of both the 1-min walk test and the standing long jump in the intervention group were close to significance (1-min walk test: 5.6 m (0.9–10.4), *p* = 0.022, standing long jump: 5.8 cm (−0.2–11.8), *p* = 0.056).

### 3.4 Sensitivity analyses

Baseline data for the sensitivity analyses are presented in Supplementary Table S4. The results of the sensitivity analyses for morphological parameters are presented in Supplementary Table S5 and results of strength and functional parameters in Supplementary Table S6, together with the results of the primary analyses. The analyses on only the most affected legs, both in the randomized and the finished group, resulted in similar coefficients and mean differences and showed no clinically relevant differences in comparison to the primary analyses including all affected legs of all participants. However, the sensitivity analyses mostly resulted in higher *p*-values due to the lower power. Both analyses indicated that including all affected legs instead of one, most affected, leg per child did not impact the results. The analyses on all randomized children showed that the change in randomization by minimization from program-driven to manual did not influence the main results, whereas the analyses on all children who finished the intervention or control group showed that the drop-out of participants due to varying reasons (see 3.1 Participants) did not impact the results.

## 4 Discussion

This study described the effects of a 12-week PRT program on muscle strength and muscle size of the lower limbs, as well as on gross motor function and walking capacity, in children with SCP, in comparison to a usual care control group. Below, we first discussed the effects of the PRT program on isometric strength, muscle morphology, and functional outcomes in relation to the literature, followed by an overview of potential explanations for the observed lack of muscle hypertrophy response, the study limitations and the final conclusion.

### 4.1 Training effect on isometric strength

The PRT program resulted in significant improvements in muscle strength in the intervention group for all trained muscle groups. This distinguished the intervention group significantly from the control group for knee flexion, and close to significantly for knee extension and plantar flexion. Overall changes were a significant 22% increase for knee extension, 97% for knee flexion and 77% for plantar flexion maximal joint torque in the intervention group, in comparison to non-significant changes of −3%, 8%, and 16% in the control group, respectively. These increases in the intervention group are in line with or larger than a previously reported average increase of 27% for targeted muscles after PRT for adolescents and young adults with SCP (Taylor et al., 2013) and increases around 10% in children with SCP (Lee et al., 2008; Scholtes et al., 2010), as well as with previous systematic reviews and meta-analyses reporting moderate to strong effects of PRT on muscle strength covering both children and adults with CP (Park and Kim, 2014; Ryan et al., 2017). The less pronounced improvement in knee extension maximal joint torque, in comparison to plantar flexion and knee flexion, might be explained because knee extension was already more targeted in usual care or by the position in which the knee extension strength was evaluated (Goudriaan et al., 2018). For children with limited knee extension range of motion and short spastic hamstring muscles, the current positioning of 30° knee flexion was close to the end of their active range of motion. PRT on the contrary focused on exercises throughout a larger range of motion (e.g., from 90° to knee extension, see Supplementary Table S1).

### 4.2 Training effect on muscle morphology

For MV, no significant interaction-effects were found, indicating no significant differences in cross-sectional muscle growth between the intervention and control group. However, muscle size increases were more than twice as large in the intervention group in comparison to the control group. The only previously performed RCT in pre-pubertal children with SCP found significant interaction-effects for rectus femoris CSA and quadriceps MT after a functional PRT (Cho and Lee, 2020). Similarly, Gillett et al. found significant increases ranging from 7.5% to 9.6% for plantar flexor MV in adolescents and adults with SCP following a 12-week functional and anaerobic strength training program, without changes in the control group (Gillett et al., 2018). On the other hand, Ryan et al. found no significant difference in the MV changes of the medial gastrocnemius in a comparison of a 10-week PRT program of the plantar flexors with usual care in 10–19 year old children and adolescents with SCP (Ryan et al., 2020).

The children in the intervention group showed significant improvements for MV of the rectus femoris (6.3%) and medial gastrocnemius (5.3%). This is much lower than the 23.1% MV increase observed for the medial gastrocnemius after 10 weeks plantar flexor training (McNee et al., 2009). It should be noted that the latter study did not include a control group, and the reported effects were larger than improvements seen in healthy adults after PRT (Schoenfeld et al., 2017b). While Lee et al. (2015) found increases in both quadriceps muscle thickness and rectus femoris CSA after 6 weeks of progressive functional training, Kruse et al. (2019) only found an increase in vastus lateralis but not rectus femoris and medial gastrocnemius muscle thickness after 8 weeks of PRT. Once the results of the current study were normalized to fibula length, mean differences in the control group became negative (ranging from 0.0 to −2.4%). In the intervention group on the other hand the results remained positive (1.2–3.7%), although only significant for the rectus femoris and close to significant for the medial gastrocnemius. The PRT program might provide possibilities to overcome the limited cross-sectional muscle growth in relation to skeletal growth seen in SCP, but with effects that were too small to be statistically significant from the control group.

In the same line as for MV, ML showed no significant interaction-effects, but increased similarly in both groups. When ML was normalized to tibia length, the mean differences in both groups were close to 0, indicating that ML followed skeletal growth. PRT had no positive nor negative effect on longitudinal muscle growth. Together with previous literature stating that PRT did not increase muscle tone or spasticity, this finding counters the notion of a potential negative influence of PRT on muscle properties for persons with spasticity (Fowler et al., 2001; Damiano et al., 2008; Scholtes et al., 2010; Stubbs and Diong, 2015; Cho and Lee, 2020). EI, on the other hand, remained unchanged for all muscles, suggesting an unaltered ratio of contractile and non-contractile muscle tissue in both groups. However, the larger standard error of measurement for EI warns us to interpret this parameter with caution (Supplementary Table S2). Only Gillett et al. (2018) estimated muscle quality after a PRT program in SCP and reported no significant difference in medial gastrocnemius intramuscular fat fraction based on MRI assessment between the intervention and control group. More specific assessments of the ratio of contractile and non-contractile muscle tissue should be used in future research to determine any changes after PRT.

### 4.3 Training effects on functional outcomes

Unilateral heel raise showed significant interaction-effects, with an improvement of 82% in the intervention group, which is in line with the results of McNee et al. (2009). Sit-to-stand and lateral step-up were close to significant for their interaction-effects and showed significant increases in the intervention group of 22% and 18%, respectively, while bilateral heel raise improved significantly in both the control and intervention group, by 17% and 22%, respectively. Scholtes et al. (2010) reported no effects on sit-to-stand and lateral step-up after a 12-week functional PRT, whereas Lee et al. (2008) found significant improvements in both sit-to-stand and lateral step-up following a 5-week training program, but only sit-to-stand was significantly different from the control group, and Gillett et al. (2018) found an overall improvement of 50% for the combination of lateral step-up, sit-to-stand and lunges in an adolescent and adult population after a combined strength and anaerobic training program. The absence of significant interaction-effects for the sit-to-stand, lateral step-up and bilateral heel raise in the current study might have different causes. First, the sit-to-stand had a smaller sample size in comparison to the unilateral tested exercises. Second, the lateral step-up and bilateral heel raise tasks require a smaller part of the maximal strength than the unilateral heel raise and sit-to-stand, respectively, and reflect a combination of balance, agility, spatial and temporal accuracy with strength endurance (Aertssen et al., 2016). Moreover, unilateral heel raises and sit-to-stand exercises were more often prescribed in the training program as they targeted the 60%–80% of 1-repetition maximum better than bilateral heel raises and lateral step-ups, which were often not intense enough. Improvements might also be related to a learning effect, which may explain improvements found in the control group.

Despite some within-group time-effects following PRT, explosive strength (evaluated with the standing long jump), walking capacity and gross motor function, did not differ between the groups. It should be noted that these outcomes were not specifically trained in the PRT program. Earlier investigations found a comparable lack of translation of PRT to gross motor function and mobility improvements (Scholtes et al., 2012; Taylor et al., 2013; Liang et al., 2021; Merino-Andrés et al., 2021). This highlights the principle of specificity of training and the need to adequately chose the type of training for the predetermined outcomes (Baker et al., 1994). A more comprehensive, functional training program might be needed to improve this carry-over (Faigenbaum and Myer, 2010). For example, training programs incorporating a power component, like the functional power training of Van Vulpen et al. (2017), and the functional and anaerobic training of Gillett et al. (2018), have shown to improve walking capacity.

### 4.4 The challenge of comparing progressive resistance training studies

There were some inconsistencies and discrepancies between the current investigation and previous muscle morphology studies (Stackhouse et al., 2007; McNee et al., 2009; Moreau et al., 2013; Williams et al., 2013; Lee et al., 2015; Gillett et al., 2018; Kruse et al., 2019; Cho and Lee, 2020; Ryan et al., 2020). First, the age of the included participants differed between studies. In previous investigations on morphological changes after PRT in patients with SCP, the ages ranged from 5 to 28 years. The current investigation can be situated at the lower end of this range. While the intervention study in older adolescents and adults showed significant increases in muscle size following PRT (Gillett et al., 2018), the results in children and younger adolescents showed inconsistencies, ranging from no to large improvements. Secondly, the training features also varied across investigations. The duration of the training program ranged from 6 to 12 weeks. While some investigations found morphological changes after 6 weeks of PRT (Lee et al., 2015; Cho and Lee, 2020), one other study did not find such effects after 10 weeks of training (Ryan et al., 2020). The applied progressive resistance training in previous studies was either isometric, analytic or functional, whereby one previous investigation combined this resistance training with anaerobic training. The training settings in previous studies were a gymnasium or rehabilitation center, at the physiotherapist, at home or a combination of these. Previous studies that applied fully home-based or combined home-based and physiotherapy-supervised interventions found limited to no results on muscle strength or hypertrophy (Kruse et al., 2019; Ryan et al., 2020), which is in contrast with the results of the current investigation. A gymnasium or rehabilitation center may provide better opportunities to maintain intensity, especially in older and more functional participants, while well-supervised home-based interventions might be practically more feasible for the parents and children. Third, inclusion criteria related to previous treatments should also be considered when comparing results of different studies. Indeed, previous studies differed regarding the timing since the last botulinum neuro-toxin A injections, as well as regarding previous lower limb bony or muscular surgery. In line with this, other differences between previous studies include the trained and investigated muscle groups, as well as the outcome parameters to define muscle size alterations, including muscle thickness, anatomical cross-sectional area and volumes of single muscles or muscle groups. Whether all muscle groups respond similarly to PRT remains to be further explored. Overall, it is challenging to generalize findings from different previous studies and define possible mechanisms regarding muscle size increases after PRT in SCP.

### 4.5 Comparison to measurement errors

Accurate assessment of morphological and functional parameters is critical both for defining the training program variables, like exercise selection, number of sets and repetitions, as well as to evaluate the efficacy of training programs (Noorduyn et al., 2011; Verschuren et al., 2011; Aertssen et al., 2018). An overview of the clinimetric properties of the used assessments (i.e., absolute and relative standard error of measurements and minimal detectable change) has been included as Supplementary Table S2. Only the improvements in knee flexion and plantar flexion isometric strength, and unilateral heel raise in the intervention group were close to or larger than their defined minimal detectable changes. Knee extension isometric strength, sit-to-stand, bilateral heel raise and standing long jump were close to or larger than their defined standard error of measurements. No morphological results were larger than the minimal detectable changes, but rectus femoris MV improvement in the intervention group as well as rectus femoris and medial gastrocnemius ML increases in both groups were close to or larger than their standard error of measurements (Noorduyn et al., 2011; Verschuren et al., 2011; Aertssen et al., 2018). The sensitivity of the applied methods and the nature of assessing children with neuromotor impairments made it difficult to detect smaller changes in some parameters. Future research should continue the search for sensitive assessments, adapted to the population and the intended intervention.

### 4.6 Potential explanations for the lack of muscle hypertrophy

Overall, the 12-week PRT program led to increased lower limb muscle strength, however, with a limited concurrent change in muscle morphology. Potential explanations for these limited muscular adaptations include a lack of mechanical injury necessary for muscle hypertrophy and a deficient hypertrophic response to the mechanical injury. Specifically, to elicit muscle hypertrophy, a net positive protein balance or anabolic state is needed where protein synthesis is larger than protein breakdown (Glass, 2005; McCarthy and Esser, 2010). This protein balance can be influenced by training and nutrition, by hormonal status and the cellular environment.

#### 4.6.1 Training stimulus

The applied PRT program was meant to provide an external training stimulus promoting the post-exercise physiological cascade and shifting the protein balance towards synthesis (Schoenfeld, 2010). The duration, volume and intensity of the PRT program were based on international guidelines for youth resistance training (Faigenbaum et al., 2009; Lloyd et al., 2014) and CP-specific training guidelines (Verschuren et al., 2011; Verschuren and Peterson, 2016). With the first weeks of training resulting in merely neural adaptations (Sale, 1988; Tesch, 1988), 12 weeks might have been too short to elicit larger adaptations in muscle mass or muscle composition. Moreover, the PRT program should result in a progressive overload, resulting from both training frequency and intensity (American College of Sports Medicine, 2009). Throughout the training period, a frequency of 3–4 training sessions per week was prescribed, taking into account expected missed training sessions due to sickness, holidays, and school trips (Scholtes et al., 2010). The median of performed training sessions was 35, representing an averaged frequency of approximately to 3 sessions per week, however with a wide range from 24 to 42. Although in adult populations an increase from 2 to 3 training sessions per week was not found to improve hypertrophy outcomes, this dose-response relationship is yet to be determined in the SCP population (Schoenfeld et al., 2016). Moreover, defining baseline strength as well as the intensity for a specific exercise remain challenging in a pediatric population. Though specific guidelines were set, the common fear for overload as well as eliciting unfavorable increases in muscle stiffness or spasticity due to PRT among physiotherapists (Bobath and Bobath, 1984) might have led to an underdosage of training intensity. Yet, to train at a sufficient intensity, it is also important to recruit as many muscle fibers as possible and expose them to the exercise stimulus (Wernbom et al., 2007). Voluntary muscle activation is decreased in children with SCP in comparison to typically developing children (Elder et al., 2003; Stackhouse et al., 2005; Mockford and Caulton, 2010) and research has indicated a limitation in the recruitment of higher-threshold motor units and in the activation of lower-threshold motor units to the same rate as typically developing children (Rose and McGill, 2005). Therefore the voluntary muscle contractions might not have produced sufficiently large forces in every participant to induce the mechanical injury necessary for muscle hypertrophy. Finally, due to the specific goals of this intervention, the training program variables and targeted muscle groups were fixed to match these goals. While, exercise selection was patient-specific, it was not possible to include patient-specific goals or outcomes. Although we tried to make the program as child-friendly as possible, mainly through the training diary which included motivational quotes, coloring pages and a training timeline where stickers could be attached after every training session, some physiotherapists and parents reported motivational issues, which may also have resulted in a limited training stimulus in some sessions. In the translation to clinical practice, the inclusion of patient specific goals or outcomes, combined with more interaction during training, e.g., through an online application or group training sessions, might promote participant motivation, as has been applied before in training studies for children with CP (Scholtes et al., 2010; Vulpen et al., 2017).

#### 4.6.2 Nutrition

Nutritional components influencing the protein balance include total calorie intake, with a specific emphasis on protein intake, as well as nutritional supplements like Vitamin-D or calcium (Verschuren et al., 2018). Although previous investigations found no difference in protein intake between typically developing children and children with SCP (Grammatikopoulou et al., 2009; Kalra et al., 2015), the timing of protein intake appears to be highest towards the evening instead of evenly balanced throughout the day (Anker-Van Der Wel et al., 2019). Moreover, it is still unclear whether children with SCP have the same protein requirements, as well as protein uptake as their typically developing peers (Verschuren et al., 2018). Especially during intensive rehabilitation programs, like PRT, the notion has been raised that protein requirements might be higher (Verschuren and Peterson, 2016). Although nutritional interventions, whether or not combined with exercise interventions, are rather limited in SCP, a recent investigation supplementing the essential amino acid leucine found both increased muscle strength (25.4%) and size (3.6%) after 10 weeks (Theis et al., 2021). Other nutritional supplements considered often in the elderly, preventing or treating sarcopenia, include Vitamin D or calcium (Verschuren et al., 2018). Research into the effects of dietary modifications and nutritional supplementations and their interaction with exercise interventions are necessary to elucidate their effects on the protein balance and consequently muscle hypertrophy in the CP population.

#### 4.6.3 Age

An additional reason for this lack of muscle hypertrophy can be found in the age of the participants, who were all pre-pubertal or early-pubertal. In a young, developing population, the adaptations should exceed the muscular strength and size increases related to growth and maturation alone. While the knowledge of the trajectory of muscle growth in children with SCP is rather limited (Verschuren et al., 2018; Williams et al., 2020), the current investigation showed non-statistically significant but clinically important negative changes in MV normalized to tibia length in the usual care control group, indicating limited cross-sectional muscle growth in relation to skeletal growth. Therefore, this population with SCP might first have to overcome this process of relative decline in muscle size before muscle growth can happen, emphasizing the importance to look at muscle size normalized to skeletal growth to determine training effects in this population. Moreover, research in typically developing children showed that strength increases during childhood, before puberty, are mainly related to the maturation of the central nervous system and therefore to improved motor unit recruitment, firing frequency, synchronization and neural myelination rather than muscular changes (Ramsay et al., 1990; Granacher et al., 2011; Lloyd et al., 2014). Strength gains later in life, during adolescence and beyond, are a combination of these neural improvements together with structural and architectural muscular changes (Fukunaga et al., 1992; Lillegard et al., 1997). The latter are a result of increased hormonal concentrations, including growth hormone, insulin-like growth factor and testosterone. Therefore, the timing of PRT in relation to the pubertal status might have to be taken into account if muscle strength improvement with concurrent muscle hypertrophy is the intended goal.

#### 4.6.4 Cellular environment

Additionally, the question should be raised whether the cellular environment of muscles in SCP has the same capacity for muscle repair, adaptation and growth as in typically developing muscles. A major factor in the post-exercise physiological cascade are satellite cells, leading to muscle regeneration from the initial mechanical injury (Murach et al., 2021). These cells are usually in a quiescent state, laying in the periphery of the muscle fiber but are triggered by external factors like exercise and injury (Snijders et al., 2015). In SCP muscles, a decreased number of satellite cells has been observed in comparison to typically developing muscles (Smith et al., 2013; von Walden et al., 2018). The influence of altered cytokine expression and extracellular matrix remodeling by fibroblasts on the complex process of muscle repair and growth, together with the alterations in satellite cell concentration and efficacy, are to be further explored in SCP muscles (Handsfield et al., 2022).

#### 4.6.5 Variability of training response

Ultimately, the response to training was quite variable. SCP is known as a very heterogeneous disorder, stemming from a range of possible brain lesions differing in timing, location and extent, and resulting in a similarly varied presentation of clinical symptoms and functional impairments (Himmelmann and Uvebrant, 2011). For example on the impairment level, muscle weakness, muscle size and the contribution of muscle size deficits to muscle weakness showed inter-individual variation (Hanssen et al., 2021). Moreover, in adult populations, PRT has been shown to cause a wide range of responses (Hubal et al., 2005; Ahtiainen et al., 2016; Bonafiglia et al., 2021). Therefore, it might be interesting for future research to identify which child might respond well to PRT based on baseline characteristics like age, gender, SMC, functional ability, topographical distribution, strength or muscle size deficits.

### 4.7 Limitations

The first limitation was the sample size and drop-outs due to Covid regulations and for other reasons. The final group size was 22 individuals, or 36 legs, in the control group and 26 individuals, or 41 legs, in the intervention group. This was just below the estimated sample size of 39–43 legs. Therefore, we were slightly underpowered for isometric and functional strength outcomes. This may explain the many interaction-effects that could be considered borderline significant. A second limitation is the inclusion of GMFCS levels, with an underrepresentation of GMFCS level II and III. The unequal distribution was mainly caused by the inclusion criteria related to previous treatments and cognitive abilities. Thirdly, strength and muscle size deficits were not used as inclusion criterium and participants with mild deficits might have had limited possibilities for improvement. On the other hand, the level of SMC was also not used as an exclusion criterium, which may have led to limitations in active and isolated contraction of the targeted muscle groups in some more affected participants. Fourth, dietary intake was not evaluated during the intervention and a lack of overall energy or protein intake could have impacted muscle hypertrophy. Finally, although the improvements in muscle strength with limited concurrent changes in muscle morphology point towards neural improvements, the exact underlying neural mechanisms were not evaluated and the underlying microscopic muscular changes remain unknown. Future research is required to elucidate both the underlying neuromuscular and cellular mechanisms to PRT, in other age groups (e.g., mid or post-puberty) with a follow-up period to evaluate the long term influence of training and detraining.

## 5 Conclusion

This study proved the effectiveness of a home-based, physiotherapy supervised PRT program to improve isometric and functional muscle strength in children with SCP without negative effects on muscle properties or any serious adverse events. Moreover, smaller but important effects were seen on maintaining or increasing muscle size. This is an accessible and applicable type of treatment for individuals with SCP. The carry-over of isometric and functional strength gains to gross motor function and walking ability was limited, highlighting the principal of specificity of training. However, the achieved strength gains could be further used to improve activity and participation in a well-planned and periodized rehabilitation program matching type of training to the training goal. Finally, the response to training remained quite variable, indicating the need to identify the baseline characteristics of responders and non-responders to PRT.
