# Supplementary material for: Progressive resistance training for children with cerebral palsy: A randomized controlled trial evaluating the effects on muscle strength and morphology
Source: Front Physiol. 2022 Oct 4;13:911162. doi: 10.3389/fphys.2022.911162 (PMC9577365; doi:10.3389/fphys.2022.911162)
Supplement: Supplementary file 1 [file Table5.pdf]

Supplementary Table 5 Estimated marginal means of mixed model analyses for muscle morphology with results for within and between analyses for the primary analyses (all participants and all affected legs (grey fill)) and the sensitivity analyses (most affected leg of all randomized participants (black) and participants who finished the control or intervention group (*grey-italic*).

Parameter		Group	n	PRE Mean (95% CI)	POST Mean (95% CI)	MEAN Δ Mean (95% CI)	Time* Group p-value	Time p-value
Muscle volume (ml)	RF	**CON**	35	64.2 (53.1-75.3)	65.8 (54.5-77.2)	1.6 (-0.1-3.4)	0.104	0.069
		**INT**	41	59.2 (48.8-69.6)	62.9 (52.2-73.5)	3.7 (1.9-5.5)		**<0.001***
		**CON**	19	63.4 (51.9-74.9)	65.1 (52.8-77.4)	1.7 (-0.7-4.0)	0.460	0.156
		**INT**	20	55.0 (44.0-66.0)	57.9 (46.0-69.8)	2.9 (0.4-5.3)		0.023
		CON	*18*	*61.9 (47.5-76.3)*	*63.4 (48.1-78.7)*	*1.5 (-0.9-3.9)*	*0.258*	*0.211*
		INT	*19*	*60.8 (47.5-74.1)*	*64.2 (49.9-78.4)*	*3.4 (1.0-5.8)*		***0.007****
	ST	**CON**	31	46.1 (38.4-53.8)	46.2 (38.4-53.9)	0.1 (-1.8-2.0)	0.322	0.931
		**INT**	38	47.0 (39.7-54.3)	48.4 (41.1-55.8)	1.4 (-0.5-3.3)		0.139
		**CON**	16	45.6 (38.1-53.2)	45.4 (37.7-53.0)	-0.3 (-3.6-3.0)	0.333	0.857
		**INT**	19	43.3 (36.1-50.5)	45.2 (37.8-52.5)	1.9 (-1.3-5.0)		0.235
		CON	*15*	*44.3 (34.4-54.1)*	*43.7 (33.8-53.5)*	*-0.6 (-3.4-2.2)*	*0.201*	*0.657*
		INT	*19*	*47.7 (39.0-56.5)*	*49.5 (40.7-58.3)*	*1.7 (-0.8-4.3)*		*0.166*
	MG	**CON**	35	43.3 (33.5-53.1)	43.8 (34.0-53.6)	0.5 (-0.5-1.6)	0.057	0.318
		**INT**	41	37.7 (28.5-46.8)	39.6 (30.4-48.8)	2.0 (0.9-3.1)		**<0.001***
		**CON**	19	41.4 (30.7-52.1)	40.9 (30.4-51.4)	-0.4 (-1.9-1.0)	**0.009***	0.538
		**INT**	20	34.5 (24.3-44.7)	36.9 (26.8-46.9)	2.4 (0.8-3.9)		**0.004***
		CON	*18*	*42.5 (30.0-54.9)*	*42.1 (29.9-54.4)*	*-0.4 (-1.7-1.0)*	*0.012*	*0.586*
		INT	*19*	*38.0 (26.5-49.4)*	*40.0 (28.8-51.2)*	*2.1 (0.7-3.4)*		***0.004****
Muscle length (mm)	RF	**CON**	33	219.8 (207.4-232.1)	225.3 (212.9-237.7)	5.5 (1.8-9.3)	0.529	**0.005***
		**INT**	40	218.9 (207.4-230.5)	222.8 (211.1-234.5)	3.9 (0.0-7.7)		0.050
		**CON**	18	220.0	226.2	6.2	0.885	0.053

				(205.7-234.3)	(211.8-240.6)	(-0.1-12.4)		
		INT	19	214.8 (201.1-228.4)	221.6 (207.6-235.6)	6.8 (-0.4-14.0)		0.061
		CON	17	214.2 (197.9-230.4)	219.7 (203.6-235.8)	5.5 (0.0-11.0)	0.962	0.049
		INT	18	221.3 (206.6-236.1)	227.1 (212.3-241.8)	5.7 (-0.1-11.5)		0.052
	ST	CON	31	215.3 (203.0-227.5)	218.2 (205.9-230.6)	3.0 (-1.6-7.5)	0.950	0.191
		INT	35	213.1 (201.4-224.7)	216.2 (204.4-228.1)	3.2 (-1.3-7.7)		0.162
		CON	16	217.0 (202.0-232.0)	218.3 (203.2-233.5)	1.3 (-4.5-7.2)	0.891	0.634
		INT	17	209.3 (194.7-223.9)	211.2 (196.5-226.0)	1.9 (-3.9-7.7)		0.505
		CON	15	213.0 (197.4-228.6)	213.3 (197.8-228.7)	0.3 (-5.4-6.0)	0.394	0.922
		INT	17	218.4 (204.4-232.3)	221.9 (208.0-235.8)	3.5 (-1.8-8.8)		0.186
	MG	CON	34	151.4 (138.4-164.4)	153.7 (140.7-166.7)	2.4 (0.3-4.4)	0.443	0.025
		INT	41	148.5 (136.3-160.6)	151.9 (139.7-164.2)	3.5 (1.4-5.6)		**0.002***
		CON	18	149.9 (135.5-164.2)	151.0 (136.6-165.4)	1.2 (-1.9-4.2)	0.303	0.440
		INT	20	144.1 (130.4-157.8)	147.5 (133.7-161.3)	3.4 (0.1-6.6)		0.042
		CON	17	152.4 (135.6-169.1)	153.5 (136.8-170.2)	1.1 (-1.5-3.7)	0.256	0.385
		INT	19	150.9 (135.6-166.2)	154.0 (138.7-169.3)	3.1 (0.6-5.7)		0.018
Echo-intensity (AU)	RF	CON	35	141.7 (134.1-149.2)	141.6 (133.9-149.3)	-0.1 (-4.2-4.1)	0.925	0.975
		INT	41	140.6 (133.6-147.7)	140.3 (132.9-147.7)	-0.3 (-4.6-3.9)		0.870
		CON	19	141.3 (132.4-150.2)	142.6 (133.4-151.8)	1.3 (-5.5-8.1)	0.688	0.702
		INT	20	141.2 (132.8-149.7)	140.6 (131.5-149.6)	-0.7 (-7.8-6.4)		0.852
		CON	18	139.6 (130.0-149.2)	141.1 (131.7-150.6)	1.5 (-4.5-7.6)	0.752	0.607
		INT	19	139.6 (130.9-148.2)	139.8 (131.2-148.4)	0.2 (-5.7-6.2)		0.940
	ST	CON	31	137.8 (128.8-146.8)	141.0 (131.8-150.2)	3.2 (-1.9-8.3)	0.859	0.218
		INT	38	133.5 (125.0-141.9)	137.3 (128.4-146.1)	3.8 (-1.1-8.7)		0.128
		CON	16	142.3 (131.8-152.8)	142.2 (131.2-153.1)	-0.1 (-8.0-7.7)	0.287	0.972
		INT	19	137.1 (127.1-147.1)	142.7 (132.0-153.3)	5.6 (-1.9-13.0)		0.139
		CON	15	140.2	140.6	0.4	0.354	0.919

				(129.0-151.4)	*(129.5-151.6)*	*(-6.7-7.4)*		
		INT	*19*	*136.5 (126.8-146.3)*	*141.2 (131.4-151.1)*	*4.7 (-1.7-11.0)*		*0.142*
	MG	**CON**	35	164.3 (158.9-169.7)	166.1 (160.6-171.6)	1.8 (-1.4-4.9)	0.496	0.267
		**INT**	41	164.8 (159.8-169.8)	165.0 (159.7-170.3)	0.2 (-3.0-3.4)		0.889
		**CON**	19	166.8 (161.1-172.5)	168.2 (162.3-174.0)	1.4 (-2.8-5.5)	0.923	0.501
		**INT**	20	165.6 (160.2-171.0)	166.7 (160.9-172.4)	1.1 (-3.2-5.4)		0.609
		CON	*18*	*165.8 (159.6-172.0)*	*166.7 (160.6-172.8)*	*1.0 (-2.7-4.6)*	*0.791*	*0.603*
		INT	*19*	*162.0 (156.4-167.6)*	*163.6 (158.1-169.2)*	*1.6 (-2.0-5.3)*		*0.369*

Primary analyses: total legs in control group=36 and in intervention group=41.
Sensitivity analyses randomized: n=20 for both groups.
Sensitivity analyses finished: n=19 for both groups.
Significant results at p<0.01 are indicated in bold and with an asterisk (*).

Abbreviations
95% CI: 95% confidence interval; CON: Control group; INT: Intervention group; MG: Medial gastrocnemius; POST: Post assessment; PRE: Baseline assessment; RF: Rectus femoris; ST: Semitendinosus.
Units
AU: Arbitrary units on 8-bit greyscale; mL: Milliliter; mm: Millimeter; n: Number.
