# Supplementary material for: Progressive resistance training for children with cerebral palsy: A randomized controlled trial evaluating the effects on muscle strength and morphology
Source: Front Physiol. 2022 Oct 4;13:911162. doi: 10.3389/fphys.2022.911162 (PMC9577365; doi:10.3389/fphys.2022.911162)
Supplement: Supplementary file 2 [file Table4.pdf]

Supplementary Table 4 Descriptive statistics of observed data at baseline for participants included in the sensitivity analyses who were randomized (black) and who finished the control or intervention group (*grey-italic*), including only the most affected side.

			CON					INT		
		n	Mean		SD		n	Mean		SD
**Isometric strength**	**KE (Nm)**	20	17.8	±	10.8		19	12.2	±	7.8
		19	*18.1*	*±*	*11.3*		*19*	*15.2*	*±*	*11.3*
	**KF (Nm)**	20	13.7	±	11.8		19	7.4	±	5.9
		19	*13.2*	*±*	*11.8*		*19*	*10.4*	*±*	*10.6*
	**PF (Nm)**	19	8.5	±	5.9		19	5.5	±	3.5
		18	*8.1*	*±*	*5.5*		*18*	*5.9*	*±*	*3.9*
**Muscle volume**	**RF (mL)**	19	68.7	±	27.6		20	59.6	±	19.8
		18	*70.4*	*±*	*27.5*		*19*	*68.3*	*±*	*29.9*
	**ST (mL)**	16	48.7	±	15.5		19	46.5	±	15.3
		15	*50.0*	*±*	*16.7*		*19*	*53.0*	*±*	*21.2*
	**MG (mL)**	19	47.6	±	27.7		20	39.3	±	15.9
		18	*51.4*	*±*	*28.2*		*19*	*44.8*	*±*	*20.1*
**Muscle length**	**RF (mm)**	18	222.8	±	33.9		19	216.0	±	31.9
		17	*222.5*	*±*	*34.9*		*18*	*228.4*	*±*	*37.0*
	**ST (mm)**	16	218.3	±	31.5		17	206.2	±	26.0
		15	*217.4*	*±*	*31.7*		*17*	*220.2*	*±*	*30.7*
	**MG (mm)**	18	154.6	±	30.3		20	147.7	±	22.6
		17	*160.4*	*±*	*31.0*		*19*	*156.1*	*±*	*26.9*
**Muscle quality**	**RF (AU)**	19	140.4	±	15.3		20	140.3	±	17.9
		18	*139.4*	*±*	*14.9*		*19*	*137.8*	*±*	*18.3*
	**ST (AU)**	16	139.9	±	17.3		19	133.8	±	19.4
		15	*138.2*	*±*	*18.8*		*19*	*133.4*	*±*	*17.3*
	**MG (AU)**	19	162.6	±	10.2		20	162.8	±	14.0
		18	*161.2*	*±*	*10.7*		*19*	*158.2*	*±*	*12.8*
**Functional strength**	**STS (n)**	19	13.3	±	5.1		19	13.4	±	4.2
		18	*13.2*	*±*	*5.4*		*19*	*14.6*	*±*	*5.5*
	**LSU (n)**	19	16.4	±	8.2		19	15.4	±	5.9
		18	*16.8*	*±*	*8.1*		*19*	*17.5*	*±*	*7.9*
	**BHR (n)**	20	23.2	±	10.5		17	21.8	±	6.0
		19	*24.1*	*±*	*10.1*		*16*	*24.5*	*±*	*10.6*
	**UHR (n)**	15	19.6	±	11.4		14	13.8	±	11.0
		15	*19.5*	*±*	*11.2*		*15*	*14.2*	*±*	*10.8*

	**SLJ (cm)**	15	83.4	±	31.0		14	67.6	±	25.2
		15	*84.3*	±	*31.2*		*14*	*76.5*	±	*30.7*
**Walking capacity**	**1MWT (m)**	19	77.9	±	25.1		17	74.4	±	17.9
		18	*81.8*	±	*21.5*		*16*	*76.1*	±	*24.0*
**Gross motor function**	**GMFM (%)**	16	81.2	±	13.0		19	77.1	±	13.4
		15	*84.0*	±	*8.3*		*19*	*79.1*	±	*16.0*

Secondary analyses randomized: n=20 for both groups.
Secondary analyses finished: n=19 for both groups.

Abbreviations
1MWT: 1-minute walk test; BHR: Bilateral heel raise; CON: Control group; GMFM: Gross motor function measure; INT: Intervention group; KE: Knee extension; KF: Knee flexion; LSU: Lateral step-up; MG: Medial gastrocnemius; PF: Plantar flexion; RF: Rectus femoris; SD: Standard deviation; SLJ: Standing long jump; ST: Semitendinosus; STS: Sit to stand; UHR: Unilateral heel raise.
Units
AU: Arbitrary units on 8-bit greyscale; cm: Centimeter; m: Meter; mL: Milliliter; mm: Millimeter; n: Number; Nm: Newton meter.
