# Supplementary material for: Progressive resistance training for children with cerebral palsy: A randomized controlled trial evaluating the effects on muscle strength and morphology
Source: Front Physiol. 2022 Oct 4;13:911162. doi: 10.3389/fphys.2022.911162 (PMC9577365; doi:10.3389/fphys.2022.911162)
Supplement: Supplementary file 3 [file Table1.pdf]

Supplementary Table 1 Some examples of training exercises with modifications

Single-joint		Multi-joint	
**Knee extensors**			
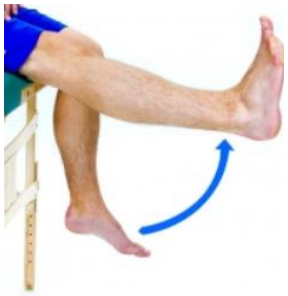	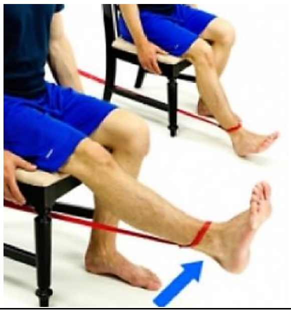	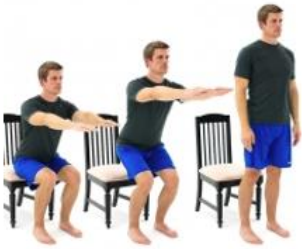	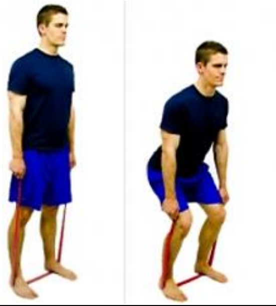
**Knee flexors**			
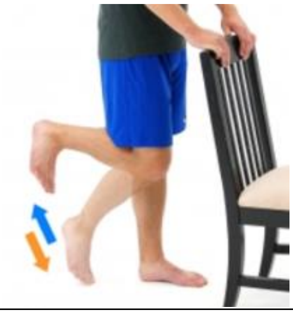	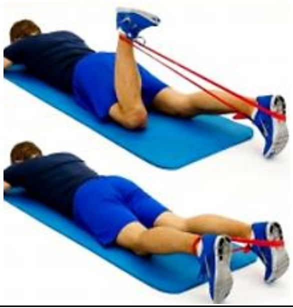	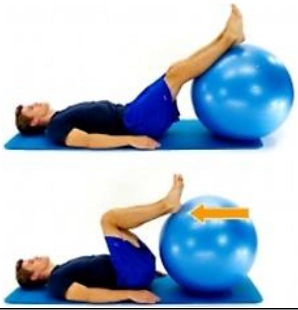	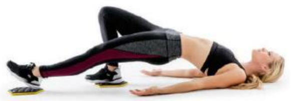
**Plantar flexors**			
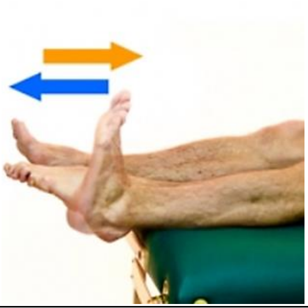	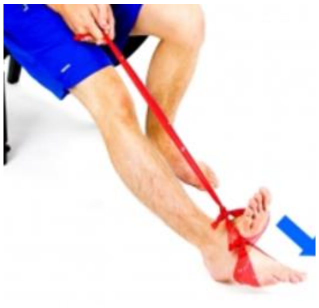	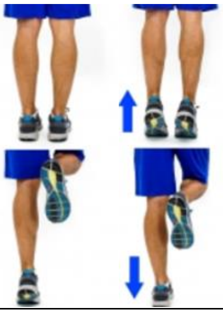	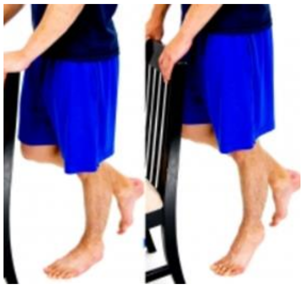

Images from https://www.hep2go.com/
