# Supplementary material for: Progressive resistance training for children with cerebral palsy: A randomized controlled trial evaluating the effects on muscle strength and morphology
Source: Front Physiol. 2022 Oct 4;13:911162. doi: 10.3389/fphys.2022.911162 (PMC9577365; doi:10.3389/fphys.2022.911162)
Supplement: Supplementary file 4 [file Table2.pdf]

Supplementary Table 2 Clinimetric properties of the applied assessments.

Parameter		ICC (95% CI)	SEM (SEM%)	MDC (MDC%)	Reference
Muscle volume (mL)	RF	0.987 (0.991-0.999)	3.5 (3.1%)	N.R.	(Hanssen et al., In press)
	ST	0.963 (0.933-0.996)	3.9 (5.1%)	N.R.	
	MG	0.979 (0.933-0.993)	8.5 (9.8%)	N.R.	
Muscle length (mm)	RF	0.983 (0.946-0.995)	5.6 (2.1%)	N.R.	
	ST	0.917 (0.670-0.983)	7.7 (2.9%)	N.R.	
	MG	0.995 (0.965-0.999)	2.0 (1.0%)	N.R.	
Echo-intensity (AU)	RF	0.866 (0.622-0.957)	5.6 (4.4%)	N.R.	
	ST	0.628 (0.002-0.864)	7.6 (5.9%)	N.R.	
	MG	0.633 (0.155-0.871)	6.6 (4.5%)	N.R.	
Isometric strength (Nm)	KE	0.935 (0.779-0.982)	3.0 (22.4%)	8.3 (62.0%)	(Verreydt et al., 2022)
	KF	0.719 (0.276-0.914)	5.6 (35.1%)	15.6 (97.3%)	
	PF	0.743 (0.193-0.936)	2.0 (30.5%)	5.6 (84.6%)	
Functional strength (n or cm)	STS	0.91 (0.80-0.96)	1.7 (18.6%)	4.8 (51.7%)	(van Tittelboom et al., 2021)
	LSU	0.92 (0.80-0.97)	3.1-3.5 (23.0-24.6%)	8.7 (63.8%)	
	BHR	0.88 (0.69-0.96)	1.9 (10.8%)	5.2 (29.8%)	
	UHR	0.98 (0.96-0.99)	0.78 (17.0%)	2.2 (48.0%)	(van Vulpen et al., 2013)
	SLJ	0.96 (0.73-0.98)	4.6^ (9.0%)	12.8 (24.9%)	(van Tittelboom et al., 2021)
Walking capacity (m)	1MWT	0.94	4.7^	13.1	(McDowell et al., 2009)
Gross motor function (%)	GMFM	0.986 (0.969-0.994)	1.9	5.3	(Brunton and Bartlett, 2011)

1MWT: 1-minute walk test; 95% CI: 95% confidence interval; BHR: bilateral heel raise; GMFM: gross motor function measure; ICC: intra-class correlation coefficient; KE: knee extension; KF:

knee flexion; LSU: lateral step-up; MDC: minimal detectable difference; MG: medial gastrocnemius; N.R.: not reported; PF: plantar flexion; RF: rectus femoris; SLJ: standing long jump; ST: semitendinosus; STS: sit-to-stand; UHR: unilateral heel raise.

^SEMs were calculated from reported MDCs based on the formula: MDC=SEM*1.96*√2.

References of studies describing the clinimetric properties of applied assessments

Brunton, L. K., & Bartlett, D. J. (2011). Validity and reliability of two abbreviated versions of the gross motor function measure. *Physical Therapy*, *91*(4), 577–588. https://doi.org/10.2522/ptj.20100279

Hanssen, B., Peeters, N., Dewit, T., Huyghe, E., Dan, B., Molenaers, G., van Campenhout, A., Bar-On, L., van den Broeck, C., Calders, P., & Desloovere, K. (In press). Reliability of 3D freehand ultrasound to assess lower limb muscles in children with spastic cerebral palsy and typical development.

McDowell, B. C., Humphreys, L., Kerr, C., & Stevenson, M. (2009). Test–retest reliability of a 1-min walk test in children with bilateral spastic cerebral palsy (BSCP). *Gait & Posture*, *29*(2), 267–269. https://doi.org/10.1016/J.GAITPOST.2008.09.010

van Tittelboom, V., Alemdaroğlu-Gürbüz, I., Hanssen, B., Plasschaert, F., Heyrman, L., Feys, H., Desloovere, K., Calders, P., & van den Broeck, C. (2021). Reliability of functional tests of the lower limbs and core stability in children and adolescents with cerebral palsy. *European Journal of Physical and Rehabilitation Medicine*. https://doi.org/10.23736/S1973-9087.21.06627-2

van Vulpen, L., de Groot, S., Becher, J. G., & de wolf, G. S. (2013). Feasibility and test-retest reliability of measuring lower-limb strength in young children with cerebral palsy. *European Journal of Physical and Rehabilitation Medicine*, *JUNE*.

Verreydt, I., Vandekerckhove, I., Hanssen, B., Stoop, E., Peeters, N., van Tittelboom, V., van den Hauwe, M., de Waele, L., van Campenhout, A., & Desloovere, K. (2022). Instrumented strength assessment in typically developing children and children with a neural or neuromuscular disorder: a reliability, validity and responsiveness study. *Frontiers in Physiology*, *Under review*.
