# Supplementary material for: Progressive resistance training for children with cerebral palsy: A randomized controlled trial evaluating the effects on muscle strength and morphology
Source: Front Physiol. 2022 Oct 4;13:911162. doi: 10.3389/fphys.2022.911162 (PMC9577365; doi:10.3389/fphys.2022.911162)
Supplement: Supplementary file 5 [file Table6.pdf]

Supplementary Table 6 Estimated marginal means of mixed model analyses for strength and functional parameters with results for within and between analyses for the primary analyses (all participants and all affected legs (grey fill)) and the sensitivity analyses (most affected leg of all randomized participants (black) and participants who finished the control or intervention group (*grey-italic*).

				**PRE**	**POST**	**MEAN Δ**	**Time* Group**	**Time**
**Parameter**		**Group**	**n**	**Mean (95% CI)**	**Mean (95% CI)**	**Mean (95% CI)**	**p-value**	**p-value**
**Isometric strength (Nm)**	**KE**	**CON**	36	14.0 (10.4-17.7)	13.6 (9.9-17.3)	-0.4 (-2.1-1.2)	0.015	0.603
		**INT**	39	11.3 (7.8-14.9)	13.9 (10.2-17.5)	2.5 (0.8-4.2)		**0.004***
		**CON**	20	14.9 (10.9-18.8)	14.7 (10.6-18.8)	-0.2 (-2.9-2.5)	0.137	0.878
		**INT**	19	9.7 (5.8-13.6)	1.4 (8.3-16.5)	2.8 (-0.2-5.7)		0.063
		CON	*19*	*14.5 (9.7-19.3)*	*14.0 (9.2-18.8)*	*-0.5 (-2.0-3.0)*	*0.086*	*0.690*
		INT	*19*	*11.6 (7.2-15.9)*	*14.1 (9.8-18.5)*	*2.6 (0.1-5.1)*		*0.046*
	**KF**	**CON**	36	10.6 (6.3-15.0)	11.4 (6.7-16.1)	0.8 (-1.9-3.4)	**0.008***	0.555
		**INT**	39	6.2 (2.0-10.4)	12.2 (7.6-16.8)	6.0 (3.3-8.7)		**<0.001***
		**CON**	20	10.9 (6.7-15.2)	11.6 (6.8-16.4)	0.7 (-2.8-4.1)	0.052	0.691
		**INT**	19	5.1 (0.9-9.3)	10.7 (5.9-15.6)	5.6 (2.0-9.3)		**0.004***
		CON	*19*	*10.5 (4.9-16.2)*	*11.7 (5.3-18.2)*	*1.2 (-2.2-4.6)*	*0.028*	*0.478*
		INT	*19*	*7.5 (2.5-12.6)*	*14.2 (8.1-20.2)*	*6.6 (3.2-10.1)*		***<0.001****
	**PF**	**CON**	35	6.9 (5.0-8.9)	8.1 (6.0-10.1)	1.1 (-0.3-2.5)	0.014	0.111
		**INT**	39	4.7 (2.8-6.6)	8.3 (6.3-10.3)	3.6 (2.2-5.0)		**<0.001***
		**CON**	19	7.4 (5.0-9.9)	8.5 (5.9-11.1)	1.1 (-3.5-1.2)	0.217	0.339
		**INT**	19	4.6 (2.2-6.9)	7.8 (5.2-10.3)	3.2 (0.8-5.6)		0.012
		CON	*18*	*6.7 (4.2-9.2)*	*7.6 (5.1-10.2)*	*1.0 (-1.1-3.0)*	*0.063*	*0.346*
		INT	*19*	*4.5 (2.2-6.8)*	*5.2 (5.9-10.4)*	*3.6 (1.6-5.6)*		***<0.001****
**Endurance functional strength (n)**	**STS**	**CON**	21	11.5 (9.5-13.5)	11.8 (9.6-14.0)	0.3 (-1.3-1.9)	0.030	0.696
		**INT**	25	12.5 (10.6-14.5)	15.2 (13.1-17.3)	2.7 (1.2-4.1)		**<0.001***
		**CON**	19	11.8	11.7	0.0	0.032	0.962

				(9.7-13.8)	(9.5-13.9)	(-1.6-1.6)		
		**INT**	19	11.9<br>(9.9-13.9)	14.4<br>(12.3-16.5)	2.5<br>(0.8-4.1)		**0.004***
		CON	*18*	*11.5*<br>*(8.8-14.2)*	*11.8*<br>*(9.1-14.5)*	*0.3*<br>*(-1.3-1.9)*	*0.043*	*0.728*
		INT	*19*	*12.9*<br>*(10.5-15.3)*	*15.5*<br>*(13.1-17.9)*	*2.5*<br>*(1.0-4.1)*		***0.002****
	**LSU**	**CON**	34	15.1<br>(11.9-18.4)	16.2<br>(12.9-19.5)	1.1<br>(-0.2-2.3)	0.076	0.099
		**INT**	38	14.8<br>(11.7-17.9)	17.4<br>(14.3-20.6)	2.7<br>(1.4-3.9)		**<0.001***
		**CON**	19	14.3<br>(10.8-17.9)	15.9<br>(12.3-19.5)	1.6<br>(-0.2-2.3)	0.486	0.071
		**INT**	19	13.7<br>(10.4-17.1)	16.2<br>(12.8-19.6)	2.4<br>(1.4-3.9)		**0.010***
		CON	*18*	*14.3*<br>*(10.3-18.4)*	*15.7*<br>*(11.7-19.7)*	*1.4*<br>*(-0.2-2.9)*	*0.211*	*0.081*
		INT	*19*	*15.3*<br>*(11.7-18.9)*	*18.0*<br>*(14.4-21.5)*	*2.7*<br>*(1.2-4.2)*		***<0.001****
	**BHR**	**CON**	22	20.9<br>(14.6-27.3)	24.5<br>(18.1-30.8)	3.6<br>(1.1-6.0)	0.288	**0.005***
		**INT**	22	20.7<br>(14.2-27.3)	25.2<br>(18.7-31.7)	4.5<br>(1.9-7.1)		**0.001***
		**CON**	20	20.4<br>(15.9-24.9)	23.8<br>(19.1-28.5)	3.4<br>(0.7-6.1)	0.532	0.016
		**INT**	17	18.0<br>(12.9-23.1)	22.6<br>(17.3-27.9)	4.6<br>(1.6-7.6)		**0.004***
		CON	*19*	*20.9*<br>*(14.6-27.3)*	*24.5*<br>*(18.1-30.8)*	*3.6*<br>*(1.1-6.0)*	*0.595*	***0.005****
		INT	*16*	*20.7*<br>*(14.2-27.3)*	*25.2*<br>*(18.7-31.7)*	*4.5*<br>*(1.9-7.1)*		***0.001****
	**UHR**	**CON**	27	17.0<br>(9.7-24.4)	15.5<br>(8.0-23.1)	-1.5<br>(-4.7-1.7)	**<0.001***	0.351
		**INT**	27	11.1<br>(2.5-19.7)	20.2<br>(11.4-28.9)	9.1<br>(5.7-12.4)		**<0.001***
		**CON**	15	17.6<br>(8.1-27.1)	17.4<br>(7.6-27.2)	-0.2<br>(-4.7-4.3)	0.019	0.930
		**INT**	14	12.4<br>(1.6-23.2)	20.2<br>(9.2-31.2)	7.8<br>(3.0-12.5)		**0.003***
		CON	*17*	*17.4*<br>*(11.2-23.6)*	*17.0*<br>*(10.9-23.2)*	*-0.3*<br>*(-4.4-3.7)*	***0.002****	*0.869*
		INT	*15*	*12.2*<br>*(5.5-18.9)*	*21.3*<br>*(14.5-28.1)*	*9.1*<br>*(5.0-13.2)*		***<0.001****
**Explosive functional strength (cm)**	**SLJ**	**CON**	16	79.8<br>(61.3-98.4)	82.6<br>(64.0-101.1)	2.7<br>(-2.8-8.3)	0.440	0.321
		**INT**	19	68.6<br>(49.8-87.3)	74.4<br>(55.5-93.3)	5.8<br>(-0.2-11.8)		0.056
		**CON**	15	79.1<br>(60.7-97.5)	83.3<br>(64.8-101.7)	4.1<br>(-1.5-9.8)	0.827	0.140
		**INT**	14	62.4<br>(44.9-79.9)	67.5<br>(49.7-85.2)	5.1<br>(-1.6-11.7)		0.129
		CON	*15*	*79.8*	*82.6*	*2.7*	*0.440*	*0.321*

				(61.3-98.4)	*(64.0-101.1)*	*(-2.8-8.3)*		
		INT	*14*	*68.6 (49.8-87.3)*	*74.4 (55.5-93.3)*	*5.8 (-0.2-11.8)*		*0.056*
**Walking capacity (m)**	**1MWT**	**CON**	21	67.2 (60.1-74.4)	70.8 (63.4-78.2)	3.6 (-1.0-8.1)	0.525	0.121
		**INT**	22	65.4 (58.4-72.5)	71.1 (63.7-78.5)	5.6 (0.9-10.4)		0.022
		**CON**	19	67.2 (59.3-75.1)	70.3 (62.2-78.5)	3.1 (-1.6-7.9)	0.572	0.189
		**INT**	17	63.2 (55.3-71.1)	68.3 (60.0-76.6)	5.1 (-0.2-10.4)		0.058
		CON	*18*	*65.6 (57.0-74.2)*	*69.0 (60.5-77.5)*	*3.4 (-1.2-8.0)*	*0.493*	*0.141*
		INT	*16*	*63.6 (55.6-71.6)*	*69.3 (61.3-77.2)*	*5.7 (0.8-10.5)*		***0.024****
**Gross motor function (%)**	**GMFM**	**CON**	18	72.6 (68.6-76.5)	72.9 (69.0-76.9)	0.3 (-1.1-1.8)	0.685	0.627
		**INT**	25	72.6 (68.8-76.4)	73.4 (69.5-77.2)	0.7 (-0.6-2.1)		0.271
		**CON**	16	72.8 (68.5-77.0)	73.3 (69.1-77.6)	0.6 (-1.1-2.3)	0.844	0.492
		**INT**	19	71.1 (66.9-75.2)	71.9 (67.6-76.1)	0.8 (-0.9-2.5)		0.339
		CON	*15*	*72.6 (67.5-77.7)*	*73.0 (67.9-78.0)*	*0.3 (-1.1-1.8)*	*0.674*	*0.652*
		INT	*19*	*72.5 (67.9-77.1)*	*73.2 (68.6-77.8)*	*0.7 (-0.6-2.1)*		*0.280*

Primary analyses: total legs in control group=36 and in intervention group=41, total participants in control group=22 and in intervention group=26.
Sensitivity analyses randomized: n=20 for both groups.
Sensitivity analyses finished: n=19 for both groups.
Significant results at p<0.01 are indicated in bold and with an asterisk (*).

Abbreviations
1MWT: 1-minute walk test; 95% CI: 95% confidence interval; CON: Control group; INT: Intervention group; BHR: Bilateral heel raise; GMFM: Gross motor function measure; KE: Knee extension; KF: Knee flexion; LSU: Lateral step-up; PF: Plantar flexion; POST: Post assessment; PRE: Baseline assessment; SLJ: Standing long jump; STS: Sit to stand; UHR: Unilateral heel raise.
Units
cm: Centimeter; m: Meter; n: Number; Nm: Newton meter; y: Years
