# Supplementary material for: Progressive resistance training for children with cerebral palsy: A randomized controlled trial evaluating the effects on muscle strength and morphology
Source: Front Physiol. 2022 Oct 4;13:911162. doi: 10.3389/fphys.2022.911162 (PMC9577365; doi:10.3389/fphys.2022.911162)
Supplement: Supplementary file 7 [file Table7.pdf]

Supplementary Table 7 Estimated marginal means of mixed model analyses for normalized strength and morphology parameters with results for within and between analyses including all participants and all affected legs.

				PRE	POST	MEAN Δ	Time* Group	Time
**Parameter**		**Group**	**n**	**Mean (95% CI)**	**Mean (95% CI)**	**Mean (95% CI)**	**p-value**	**p-value**
**Normalized isometric strength (Nm/kg)**	**KE**	**CON**	36	0.50 (0.39-0.60)	0.48 (0.37-0.59)	-0.02 (-0.08-0.04)	0.048	0.525
		**INT**	40	0.43 (0.33-0.53)	0.50 (0.40-0.61)	0.07 (0.01-0.13)		0.032
	**KF**	**CON**	36	0.36 (0.25-0.47)	0.39 (0.28-0.51)	0.03 (-0.04-0.11)	**0.005***	0.379
		**INT**	39	0.22 (0.11-0.32)	0.41 (0.29-0.52)	0.19 (0.11-0.27)		**<0.001***
	**PF**	**CON**	35	0.25 (0.18-0.32)	0.29 (0.22-0.36)	0.04 (-0.01-0.09)	0.013	0.117
		**INT**	39	0.19 (0.12-0.26)	0.32 (0.25-0.39)	0.13 (0.08-0.18)		**<0.001***
**Normalized muscle volume (ml/cm)**	**RF**	**CON**	35	2.33 (2.03-2.62)	2.32 (2.02-2.62)	0.00 (-0.06-0.06)	0.041	0.941
		**INT**	41	2.14 (1.87-2.42)	2.22 (1.94-2.51)	0.08 (0.02-0.14)		**0.007***
	**ST**	**CON**	31	1.68 (1.48-1.88)	1.64 (1.44-1.84)	-0.04 (-0.10-0.03)	0.263	0.287
		**INT**	38	1.69 (1.50-1.88)	1.71 (1.51-1.90)	0.02 (-0.05-0.08)		0.615
	**MG**	**CON**	35	1.54 (1.28-1.81)	1.53 (1.26-1.80)	-0.02 (-0.05-0.02)	0.074	0.450
		**INT**	41	1.37 (1.12-1.62)	1.40 (1.15-1.65)	0.04 (-0.01-0.08)		0.085
**Normalized muscle length (cm/cm)**	**RF**	**CON**	33	0.81 (0.79-0.84)	0.81 (0.79-0.84)	0.00 (-0.01-0.02)	0.780	0.950
		**INT**	40	0.81 (0.79-0.83)	0.81 (0.79-0.83)	0.00 (-0.02-0.01)		0.745
	**ST**	**CON**	31	0.80 (0.77-0.82)	0.79 (0.76-0.82)	-0.01 (-0.02-0.01)	0.417	0.432
		**INT**	35	0.79 (0.76-0.82)	0.79 (0.76-0.82)	0.00 (-0.01-0.02)		0.715
	**MG**	**CON**	34	0.56 (0.53-0.59)	0.50 (0.53-0.58)	-0.01 (-0.04-0.03)	0.648	0.740
		**INT**	41	0.55 (0.53-0.58)	0.55 (0.52-0.58)	0.00 (-0.04-0.03)		0.879

Total legs in control group=36 and in intervention group=41. Significant results at p<0.01 are indicated in bold and with an asterisk (*).

Abbreviations
95% CI: 95% confidence interval; CON: Control group; INT: Intervention group; KE: Knee extension; KF: Knee flexion; MG: Medial gastrocnemius; PF: Plantar flexion; POST: Post assessment; PRE: Baseline assessment; RF: Rectus femoris; ST: Semitendinosus.

Units<br>cm/cm: Centimeter/centimer; mL/cm: Milliliter/centimeter; n: Number; Nm/kg: Newton-meter/kg
