# Supplementary material for: Progressive resistance training for children with cerebral palsy: A randomized controlled trial evaluating the effects on muscle strength and morphology
Source: Front Physiol. 2022 Oct 4;13:911162. doi: 10.3389/fphys.2022.911162 (PMC9577365; doi:10.3389/fphys.2022.911162)
Supplement: Supplementary file 6 [file Table3.pdf]

Supplementary Table 3 Descriptive statistics of patient characteristics for participants included in the sensitivity analyses, i.e. participants who were randomized and participants who finished the control or intervention group.

	Randomized							Finished					
	**CON (20)**			**INT (20)**				**CON (19)**			**INT (19)**		
	Frequencies (%)			Frequencies (%)				Frequencies (%)			Frequencies (%)		
**GMFCS**													
Level I	14 (70.0)			12 (60.0)				13 (68.4)			12 (63.2)		
Level II	3 (15.0)			5 (25.0)				5 (26.3)			4 (21.0)		
Level III	3 (15.0)			3 (15.0)				1 (5.3)			3 (15.8)		
**Age group**													
<8 years	11 (55.0)			11 (55.0)				11 (57.9)			9 (47.4)		
>8 years	9 (45.0)			9 (45.0)				8 (42.1)			10 (52.6)		
**Involvement**													
Unilateral	7 (35.0)			9 (45.0)				8 (42.1)			9 (47.4)		
Bilateral	13 (65.0)			11 (55.0)				11 (57.9)			10 (52.6)		
**Gender**													
Boy	14 (70.0)			9 (45.0)				16 (84.2)			8 (42.1)		
Girl	6 (30.0)			11 (55.0)				3 (15.8)			11 (57.9)		
	**Mean**		**SD**	**Mean**		**SD**		**Mean**		**SD**	**Mean**		**SD**
**Age (years)**	8.3	±	2.2	7.8	±	1.6		8.2	±	2.1	8.4	±	2.1
**Weight (kg)**	27.8	±	7.3	26.6	±	7.9		28.3	±	7.7	28.8	±	8.9
**Height (cm)**	128.1	±	11.7	123.8	±	11.8		127.8	±	11.9	129.0	±	14.8
**Fibula (mm)**	27.6	±	3.4	26.8	±	3.1		27.7	±	3.6	28.0	±	3.6
**SCALE**	6.9	±	2.5	6.7	±	1.9		7.5	±	2.1	6.7	±	2.1

Total participants in both groups that were randomized: n=20.
Total participants in both groups that finished: n=19.
Abbreviations
CON: Control group; GMFCS: Gross motor functional classification system; INT: Intervention group; N: Number; SCALE: Selective Control Assessment of the Lower Extremity; SD: Standard deviation.
Units
cm: Centimeter; kg: Kilogram; mm: Millimeter.
